# Waxy‐ or Putty‐Like Materials as a Novel Drug Preparation for Synthetic Cannabinoid Receptor Agonists: Detection in Prisons and In Vitro Cannabinoid Receptor Activity

**DOI:** 10.1002/dta.3817

**Published:** 2024-10-15

**Authors:** Axelle Timmerman, Marie H. Deventer, Rachael Andrews, Robert Reid, Victoria Marland, Darren Edwards, Christopher R. Pudney, Niamh Nic Daéid, Christophe P. Stove, Caitlyn Norman

**Affiliations:** ^1^ Laboratory of Toxicology, Department of Bioanalysis, Faculty of Pharmaceutical Sciences Ghent University Ghent Belgium; ^2^ Department of Life Sciences University of Bath Bath UK; ^3^ Leverhulme Research Centre for Forensic Science, School of Science and Engineering University of Dundee Dundee UK; ^4^ Drug Discovery Unit, School of Life Sciences University of Dundee Dundee UK; ^5^ Centre for Bioengineering and Biomedical Technologies University of Bath Bath UK; ^6^ Department of Biomedical and Clinical Science, Division of Clinical Chemistry and Pharmacology Linköping University Linköping Sweden

**Keywords:** CB_1_ receptor bioassay, MDMB‐INACA, new psychoactive substances (NPS), novel drug preparations, synthetic cannabinoid receptor agonists (SCRAs)

## Abstract

After the Scottish Prison Service (SPS) introduced mail photocopying procedures in December 2021, a shift in smuggling methods was observed for synthetic cannabinoid receptor agonists (SCRAs) and other new psychoactive substances (NPS) from drug‐infused papers back to traditional sample matrices (e.g., tablets and powders), although new matrices also emerged. This study reports on waxy‐ or putty‐like materials as a novel drug preparation for SCRAs and other drugs seized from UK prisons. In 2023, 22 of these new preparations were seized from Scottish prisons, with eight found in sealed vape pods. The materials were positive for SCRAs, phytocannabinoids, novel benzodiazepines, and/or gabapentinoids. Additionally, 11 preparations were seized from an English prison, all containing the SCRAs MDMB‐4en‐PINACA and MDMB‐INACA. MDMB‐INACA was pharmacologically characterized using in vitro CB_1_ and CB_2_ bioassays, revealing moderate efficacy but low potency at CB_1_. Furthermore, the in vitro CB_1_ bioassay was also used to evaluate the CB_1_ activating potential of extracts from eight seized samples. Six of these showed high CB_1_ activity, whereas the samples lacking SCRAs or containing only MDMB‐INACA showed no or only weak CB_1_ activity, respectively. Lastly, applying the bioassay as an activity‐based “untargeted” screening method effectively identified the presence of SCRAs in one waxy preparation, which was initially not detected by gas chromatography–mass spectrometry (GC‐MS). This underscores the effectiveness of the bioassay for evaluating these new waxy‐ or putty‐like materials for the presence of SCRAs.

## Graphical abstract

Waxy‐ or putty‐like materials has emerged as a novel drug preparation for synthetic cannabinoids and other drugs in UK prisons. An in vitro CB_1_ bioassay was used to evaluate the CB_1_ activating potential of MDMB‐INACA and extracts from eight seized samples. MDMB‐INACA had moderate efficacy but low potency at CB_1_.

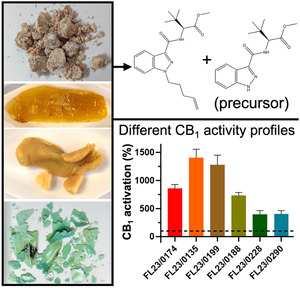

## 1 Introduction

Synthetic cannabinoid receptor agonists (SCRAs), colloquially known as “spice,” are one of the largest and most diverse classes of new psychoactive substances (NPS) with over 250 different compounds detected in Europe to date [1]. These compounds aim to mimic the psychoactive effects of Δ^9^‐tetrahydrocannbinol (Δ^9^‐THC), the main psychoactive component of cannabis, by targeting the cannabinoid 1 (CB_1_) receptor. However, most of them also interact with the cannabinoid 2 (CB_2_) receptor, which is mainly located on cells involved in the immune system [2, 3]. The potency and efficacy of SCRAs can vary widely between compounds, with many being much more potent and efficacious than Δ^9^‐THC [3, 4]. As a result, these compounds are frequently linked to several adverse effects such as seizures, tachycardia, anxiety, hallucinations, coma, and even death [5, 6].

**FIGURE 1:**
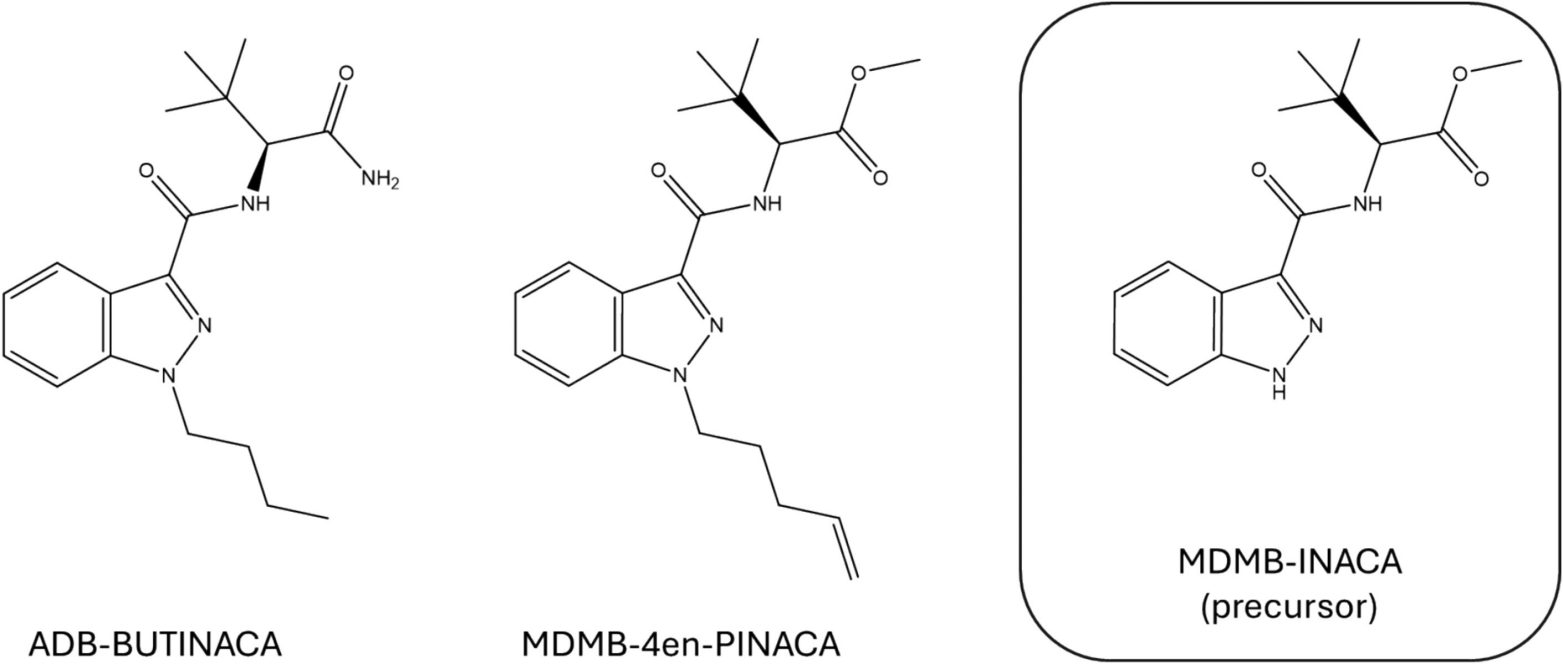
Chemical structures of ADB‐BUTINACA and MDMB‐4en‐PINACA as main SCRAs identified in this study, and the precursor MDMB‐INACA.

The SCRA market is highly dynamic and constantly evolving, often in response to current national and international legislation. Although there is a wide structural variety in compounds [1], many SCRAs have a chemical structure that can be categorized into four parts: a core, linker, head, and tail group [7]. Based on these shared SCRA features, generic legislations such as the 2021 class‐wide regulation in China, known as a major producing country of SCRAs and their precursors, have been introduced to regulate all structurally related analogs of common SCRA scaffolds [8]. However, these regulatory measures also encourage the synthesis of “atypical” SCRA features designed to circumvent current legislations. In 2021, the tail‐less indazole‐based compound MDMB‐INACA, carrying a *tert*‐leucine methyl ester (MDMB) head group (Figure 1), entered the SCRA market and has since been detected in Europe [9, 10], the United States [11], and New Zealand [12]. It is closely related to ADB‐INACA, an analog carrying a *tert*‐leucinamide (ADB) head group, and ADB‐5′Br‐INACA, a bromine‐substituted derivative, both detected in the United States and Europe in 2022 [13, 14]. For these substances, removal of the alkyl tail moiety enabled them to bypass the Chinese generic legislation.

These tail‐less SCRAs may be considered precursors in the synthesis of controlled SCRAs. The most common approach to SCRA synthesis has the alkylation of the core first followed by the coupling reaction with the head moiety, so the tail‐less SCRA is not a precursor and does not appear as an impurity. However, there is another synthesis route that has the alkylation of the core (addition of the tail) as the final step, meaning the tail‐less SCRAs are precursors [15]. Tail‐less SCRAs have been found for sale on the surface web as part of “Do‐It‐Yourself (DIY) kits” for the synthesis of controlled SCRAs, such as MDMB‐4en‐PINACA. These kits provide “semi‐finished” compounds or precursors alongside other reaction components, including a halogenated tail (e.g., 1‐bromobutane or 5‐bromo‐1‐pentene), and instructions to synthesize the intended SCRA [16]. Screenshots from websites where these kits were found for sale are provided in the Supporting Information (Section S1). This new method of purchasing tail‐less SCRAs as precursors and synthesizing controlled SCRAs through alkylation of the core would explain the only recent detection of tail‐less SCRAs in SCRA seizures following the enactment of the Chinese generic legislation.

SCRA use has been found to be a large problem within prisons in many countries [17, 18, 19, 20, 21, 22]. SCRAs and other drugs, mainly NPS (e.g., novel benzodiazepines and novel synthetic opioids), are often smuggled into prisons via the mail system as infused papers [23, 24, 25, 26, 27, 28]. These infused papers are typically rolled into cigarettes for smoking, but the heterogeneous drug concentrations across the sheets of paper lead to inconsistent dosing. However, within Scottish prisons, the implementation of a smoking ban at the end of 2018 resulted in prisoners vaping infused papers instead [23]. A similar shift from smoking to vaping drugs was observed in English prisons following the implementation of their smoking ban in 2017 [29]. In December 2021, in response to the rise in drug‐infused papers, the Scottish Prison Service (SPS) implemented mail photocopying procedures. This involved photocopying of the original papers, potentially containing drugs, with the photocopies then given to the prisoners [30]. This new policy resulted in a shift back to traditional sample matrices, mainly powders and tablets, but newer matrices have also begun to emerge, including drug‐infused clothing and waxy‐ or putty‐like materials [27]. The latter is reminiscent of the cannabis concentrates known as butane hash/honey oil (BHO), “shatter,” “budder,” “wax,” and so forth. [31, 32, 33, 34, 35, 36]. The fact that neither the concentration nor the nature of the specific SCRA(s) used in these newly detected waxy‐ or putty‐like materials are known poses an increased risk of intoxication and unanticipated effects.

To the best of the authors' knowledge, this is the first report in the literature on waxy‐ or putty‐like materials as a novel drug preparation for SCRAs and other drugs (e.g., novel benzodiazepines and gabapentinoids) in Scottish and English prisons. Furthermore, using activity‐based bioassays, the in vitro intrinsic CB_1_ receptor activation potential present in these new drug preparations was determined and the new tail‐less SCRA MDMB‐INACA was for the first time pharmacologically characterized at CB_1_ and CB_2_.

## 2 Materials and Methods

### 2.1 Materials

#### 2.1.1 Seized Samples (Dundee, UK)

The samples included in this study were non‐attributable samples seized between January 31 and October 12, 2023 by the SPS. Samples were individually sealed in labeled tamperproof evidence bags and, once deemed suitable for analysis, anonymized by SPS staff and delivered by Police Scotland to the Home Office licensed drug testing laboratory at the Leverhulme Research Centre for Forensic Science (LRCFS) at the University of Dundee.

Liquid chromatography‐mass spectrometry (LC‐MS) grade methanol (MeOH), water, and acetonitrile (ACN) were purchased from Fisher Scientific, UK; bupivacaine and formic acid were obtained from Sigma‐Aldrich (Poole, UK). The MDMB‐INACA reference standard (≥98% purity) was obtained from Cayman Chemical (Ann Arbor, MI, USA). ADB‐BUTINACA (>98% purity) and MDMB‐4en‐PINACA (98.6% purity) were synthesized and supplied by the Sutcliffe Group at Manchester Metropolitan University, Manchester, UK, as described previously [37, 38]. Reference standards were supplied as powders and then prepared as 100 or 200 μg/mL solutions in MeOH for analysis.

#### 2.1.2 Seized Samples (Bath, UK)

Eleven drug samples were obtained from an English prison, all of which were seized between March 6 and August 29, 2023, following an attempted throwover (thrown over the prison boundary). Samples were sealed in eight labeled tamperproof evidence bags, three of which contained two separate samples wrapped in cling film. All samples were anonymized and delivered to the University of Bath.

LC‐MS grade water was obtained from Merck (Watford, UK), MeOH (LC‐MS grade) from VWR (Lutterworth, UK), formic acid from Honeywell Fluka (Bracknell, UK), and ACN (LC‐MS grade) and maleic acid from Sigma‐Aldrich.

#### 2.1.3 Determination of In Vitro CB_1_ and CB_2_ Receptor Activity (Ghent, Belgium)

Dulbecco's Modified Eagle's Medium (DMEM) (GlutaMAX™), Opti‐MEM® I Reduced Serum, amphotericin B, and penicillin/streptomycin (10,000 IU/mL and 10,000 μg/mL) were purchased from Thermo Fisher Scientific (Waltham, MA, USA). The Nano‐Glo® Live Cell reagent and the corresponding Nano‐Glo® LCS Dilution buffer were obtained from Promega (Madison, WI, USA). Fetal bovine serum (FBS) and poly‐D‐lysine were purchased from Sigma‐Aldrich (Darmstadt, Germany). MeOH was obtained from Chem‐Lab NV (Zedelgem, Belgium). The reference standards for MDMB‐INACA (purity ≥98%) and (−)‐CP55,940 (purity ≥98%) were from Cayman Chemical and JWH‐018 was procured from LGC (Wesel, Germany).

### 2.2 Methods

#### 2.2.1 Seized Sample Analysis (Dundee, UK)

For the analysis of waxy‐ or putty‐like materials, the material was weighed (or dried and then weighed in the event of liquid content) and then approximately 10 mg of the material was extracted in 0.5 mL of 0.25 mg/mL bupivacaine in MeOH by ultrasonication (5 min). For sealed vape pods, 1 mL of 0.25 mg/mL bupivacaine in MeOH was pipetted down the mouthpiece into a beaker and then the entire vape pod was sonicated (5 min) in the solution. Following analysis, the vape pods were opened using pliers to remove the heating element and the waxy‐ or putty‐like materials inside were removed and weighed.

Sample extracts were analyzed by GC‐MS using a 7820A gas chromatograph coupled to a 5977E mass spectrometer (Agilent Technologies, Santa Clara, CA, USA). Injection mode: 1‐μL sample injection was used with a 20:1 split into a 4‐mm internal diameter deactivated glass liner pre‐packed with quartz wool, injection port temperature: 200°C, carrier gas: He, flow: 1 mL/min. Column: HP‐5MS, 0.33 μm, 0.2 mm × 25 m (Agilent Technologies). GC oven: 80°C held for 3 min; 40°C/min to 300°C, held for 6 min; total run time: 14.5 min; transfer line: 295°C. The mass spectrometer operated in electron impact ionization (EI) mode. Ionization conditions: 70 eV in full scan mode (50–550 amu), ion source: 230°C, quadrupole: 150°C.

Compounds were identified by comparing the GC‐MS retention times and mass spectra for the seized samples to that of the reference standards analyzed within 24 h of the sample under the same instrumental conditions. The sample GC retention time had to fall within 0.05 min of the retention time of the appropriate reference standard to be considered a match. If samples had high concentrations of the compound (as assessed by peak area), causing peak shape distortion and shifting of peak apex, the sample GC retention time had to fall within 0.1 min of the reference standard.

Prior to obtaining a reference standard, the detection of MDMB‐INACA in some samples was confirmed by analyzing the dilutions of the sample extracts (1:100 v:v) using ultra‐high performance liquid chromatography coupled to time‐of‐flight mass spectrometry (UPLC‐QToF‐MS). The UPLC‐QToF‐MS analysis was performed using an Acquity UPLC instrument with a binary pump, autosampler held at 4°C, vacuum degasser, and a column oven held at 30°C coupled to a Xevo QToF‐MS (Waters Corporation, Milford, MA, USA). The mobile phases used were (A) LC‐MS grade water with 0.1% formic acid and (B) ACN with 0.1% formic acid. The gradient used was 50:50 A:B from 0.0 to 4.0 min, 25:75 A:B from 4.0 to 5.0 min, 5:95 A:B from 5.0 to 5.99 min, and 50:50 A:B from 6.0 to 7.0 min. The flow rate was 0.5 mL/min and 2 μL of the sample was injected onto a BEH C_18_ 50 × 2.1 mm, 1.7 mm particle size column (Waters Corporation). The QToF was operated in positive ionization mode with a source temperature of 120°C, a desolvation temperature at 500°C, and a capillary voltage at 2.25 kV. ToF‐MS analysis for the high‐resolution determination of molecular mass was carried out with a collision energy at 6 V. The MS^e^ acquisition was performed using collision energies ranging from 0 to 40 V. After the QToF‐MS and MS^e^ data were processed, accurate parent ion fragmentation spectra were obtained using MS/MS data acquisition of the selected parent ion accurate mass data using collision energies between 10 and 30 V.

For samples where a mixture of compounds was present, the composition of the samples was estimated using the percentage total peak area for each compound. The percentage total peak area was determined by comparing the peak areas of each compound to the total peak area of all active components in the sample. The percentage peak area was then corrected to account for the different EI‐MS detector response of each compound by analyzing the samples alongside a mixture prepared from reference materials at the same concentrations (100 μg/mL). Based on a comparison of the peak areas of the reference materials, correction factors were calculated and applied to the peak areas of each compound in a sample (see Table S2.1).

#### 2.2.2 Seized Sample Analysis (Bath, UK)

For LC‐QToF‐MS analysis, all samples were weighed, dissolved in MeOH at a concentration of 1 mg/mL, and diluted to 1 μg/mL. Analyses were performed using an Agilent QToF 6545 with a Jetstream electrospray ionization (ESI) source coupled to an Agilent 1260 Infinity II Quaternary pump HPLC with a 1260 autosampler, column oven compartment, and variable wavelength detector (VWD). The mobile phases used were (A) LC‐MS grade water with 0.1% formic acid and (B) ACN with 0.1% formic acid. The gradient used was 70:30 A:B from 0.0 to 3.0 min, 0:100 A:B from 3.0 to 5.6 min, and 70:30 A:B from 5.6 to 7.6 min. The flow rate was 0.5 mL/min at 50°C, and 5 μL of the sample was injected onto an EC‐C_18_ 3.0 × 50 mm, 2.7‐μm particle size column (InfinityLab Poroshell 120, Agilent Technologies). The MS was operated in positive ionization mode with the gas temperature at 250°C, the drying gas at 11 L/min, and the nebulizer gas at 35 psi (2.41 bar). The sheath gas temperature was set to 300°C, and the flow rate was 12 L/min. The MS was calibrated using a reference calibrant introduced from an independent ESI reference sprayer. The VCap, Fragmentor, and Skimmer were set to 3500, 160, and 45 V, respectively. The MS was operated in all‐ions mode with three collision energy scan segments at 0, 20, and 40 eV.

The VWD was set to detect at 280‐nm wavelength at a frequency of 2.5 Hz. Data processing was automated in Qual 10, with the molecular feature extraction set to the largest 20 compounds for [M + H]^+^, [M‐H]^−^, and [M + HCOO]^−^ ions. The results were also searched against the online mass spectral database HighResNPS [39, 40] (containing over 2300 unique compound entries) with a forward score of 25 and reverse score of 70 and mass tolerances within 5 ppm of the reference library matches. Qualified ions had co‐elution scores of ≥90, retention time tolerances of ±0.10, and a minimum S/N of ≥5.00.

For quantitative nuclear magnetic resonance (NMR), 10 mg of the seized material was mixed with approximately 5‐ to 10‐mg maleic acid, which was used as an internal standard. Quantitative NMR was carried out by recording accurate masses of both the drugs in the seized sample and the internal standard maleic acid using a four‐decimal place analytical balance. ^1^H NMR data were determined at 500 MHz in CD_3_OD and chemical shifts were reported downfield from tetramethylsilane (TMS). NMR spectra were recorded on a 500 MHz ProPulse spectrometer (Agilent Technologies) with a 96‐position sample changer. The spectra were compared with the NMR data in the literature. The following equation, adapted from Naqi et al. [41], was used for the ^1^H qNMR quantitation:

$$P\;\left[\%\right]=\frac{n_{\mathrm{IC}}\cdot {\mathrm{Int}}_{x}\cdot {\mathrm{MW}}_{x}\cdot m_{\mathrm{IC}}}{n_{x}\cdot {\mathrm{Int}}_{\mathrm{IC}}\cdot {\mathrm{MW}}_{\mathrm{IC}}\cdot m_{s}}\cdot P_{\mathrm{IC}}$$

where *P* was the purity, *n* was the number of protons, *Int* was the integral value, *MW* was the molecular weight, *m* was the mass, IC was the internal calibrant, *x* was the analyte, and *s* was the sample. As multiple compounds were present in each sample, only nonoverlapping NMR peaks were used for quantitation calculations (7.59 ppm for MDMB‐INACA and 5.84, 7.29, 7.46, 7.63, and 8.20 ppm for MDMB‐4en‐PINACA).

#### 2.2.3 Analysis by LC‐QToF‐MS (Ghent, Belgium)

Sample FL23/0290 was examined by LC‐QToF‐MS analysis. Chromatographic separation was performed with an Agilent 1290 Infinity LC system coupled to a Phenomenex Kinetex C18‐column (2.6 μm, 3 mm × 50 mm), maintained at 30°C. The high‐resolution mass spectrometry (HRMS) system was a 5600 + QTOF from Sciex (Framingham, MA, USA) with an ESI source (positive mode) and Sciex Analyst TF 1.8.1 software being used to manage the system. The mobile phases used were (A) 0.05% formic acid and 5‐mM ammonium formate in LC‐MS grade water and (B) 0.05% formic acid in 50:50 MeOH:ACN. The settings for the LC‐HRMS were the same as those previously published [42, 43], with an LC gradient starting at 50:50 A:B, with a linear increase to 2:98 A:B in 5 min. This resulted in TOF‐MS full scan spectra (scanning from 250 to 500 Da) combined with data dependent acquisition of product ion spectra (scanning from 50 to 500 Da). Ten microliters μL of a 1:1000 dilution of the initial methanolic extract in diluent (12.5% 50:50 ACN/MeOH in water) was injected.

#### 2.2.4 Determination of In Vitro CB_1_ and CB_2_ Receptor Activity (Ghent, Belgium)

The intrinsic activation potential of MDMB‐INACA at both CB_1_ and CB_2_ receptors was determined using live cell‐based reporter assays, monitoring β‐arrestin 2 (βarr2) recruitment via the NanoLuc Binary Technology (NanoBiT®). For extracts derived from waxy‐ or putty‐like material, the CB_1_ activation potential was evaluated using the CB_1_ reporter system. Details on the generation of the reporter systems have been previously described [44, 45, 46]. Human embryonic kidney (HEK) 293T cells stably expressing the CB_1_‐βarr2 or CB_2_‐βarr2 system were routinely maintained at 37°C and 5% CO_2_ in a humidified atmosphere and cultured in DMEM (GlutaMAX™) supplemented with 10% heat‐inactivated FBS, amphotericin B (0.25 μg/mL), penicillin (100 IU/mL), and streptomycin (100 μg/mL). The experiments were performed following a two‐day protocol. On the day prior to the experiment, cells were seeded in poly‐D‐lysine coated 96 well‐plates at a density of 5 × 10^4^ cells/well and incubated overnight. On the day of the assay, the cells were rinsed twice with Opti‐MEM® I and 100 μL of this medium was added to each well. Subsequently, the Nano‐Glo® Live cell reagent was prepared according to the manufacturer's protocol (i.e., diluted 1/20 in the Nano‐Glo® LCS Dilution buffer) and 25 μL of this substrate mix was added to each well. The plate was then placed in the TriStar^2^ LB 942 Multimode Microplate Reader (Berthold Technologies GmbH & Co., Germany) and luminescence was recorded for approximately 10 min (initial equilibration phase). Upon stabilization of the signal, 10 μL of a 13.5× concentrated test solution was added and luminescence was monitored for 2 h. The test solutions were prepared within 24 h before the experiment in Opti‐MEM® I/MeOH (50:50 v/v). A concentration range of CP55,940 was included on every plate as a reference standard and was later used for normalization of the data. The prototypical SCRA JWH‐018 was analyzed in tandem for easier comparison with earlier reported SCRA activity data. Appropriate solvent controls were present on each plate. For analysis of the extracts from the waxy‐ or putty‐like material, methanolic extracts (see sample preparation) were 100‐fold diluted in MeOH and further diluted 1:1 in Opti‐MEM® I. Ten microliters of this diluted extract was then analyzed by the bioassay. A solvent control consisting of MeOH/Opti‐MEM® I (50:50 v/v), with a final *in‐well* concentration of 3.7% MeOH, was included on each plate.

For the characterization of MDMB‐INACA, raw luminescence data were processed using Microsoft Excel 2019. These values were corrected for *interwell* variability (using luminescence data obtained during the equilibration phase) and the area under the curve (AUC) values were calculated, which were then subjected to a blank correction by subtracting the AUC values of the corresponding solvent controls. Corrected data were further processed using GraphPad Prism Software (version 10.0.0) and normalized to the *E*_max_ of CP55,940, arbitrarily set at 100%. Potency (*EC*_50_) and efficacy (*E*_max_) were determined for MDMB‐INACA via curve fitting of the normalized concentration response curves (nonlinear regression, three‐parameter logistic fit). Each datapoint is represented as AUC ± standard error of mean (SEM) from at least three independent experiments, run in duplicate. Datapoints for the highest concentrations were excluded from the dataset in the case of a reduction of >20% in the highest concentration compared with the following concentration. The Grubbs test (*p‐*value <0.05) was used to scan for potential outliers, which were then omitted from the dataset (applicable for 1 out of 339 datapoints for CP55,940, MDMB‐INACA, and JWH‐018). For the evaluation of CB_1_ activity in the waxy‐ or putty‐like materials, *interwell* corrected luminescence signals were used and represented as relative light unit (RLU) ± SEM from three independent experiments and compared with the solvent control (Opti‐MEM® I/MeOH [50:50 v/v]).

## 3 Results and Discussion

### 3.1 Seized Sample Analysis

**FIGURE 2:**
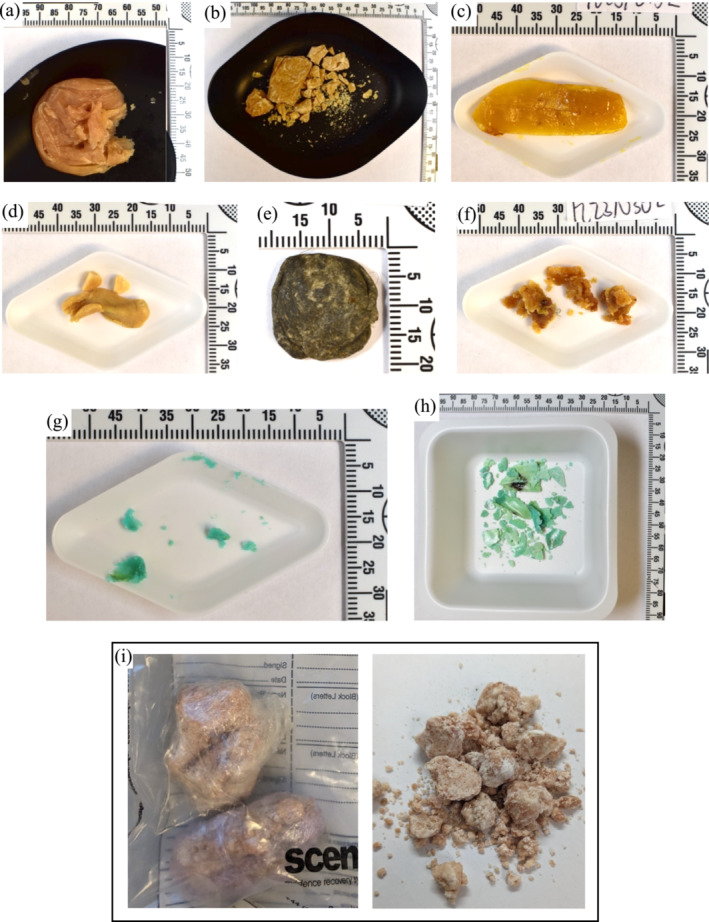
Examination photographs of samples of waxy‐ or putty‐like materials seized from the Scottish prisons: (a) FL23/0180‐1, (b) FL23/0174, (c) FL23/0152, (d) FL23/0301, (e) FL23/0367, (f) FL23/0302, (g) FL23/0167 at first examination, (h) FL23/0167 at second examination following exposure to air/evaporation, and (i) examination photographs of a sample seized from the English prisons.

Between January 3 and October 12, 2023, 14 samples consisting of waxy‐ or putty‐like materials were seized from four Scottish prisons. Details of the samples can be found in Table 1 (sample set 1) and full analytical details are available in the Supplementary Information (Table S3.1). Examination photographs of these materials that demonstrate the variety of sample consistencies/textures can be found in Figure 2 and a complete set of photographs is available in the Supporting Information (Figures S7.1–S7.3). Most of these samples were found wrapped in cling film or in plastic sachets. The samples weighed between 0.017 and 5.24 g (mean: 1.34 g, median: 0.74 g), and 11 were found to contain the SCRAs ADB‐BUTINACA and/or MDMB‐4en‐PINACA. Remarkably, the majority also contained MDMB‐INACA, present at between 3.30% and 81.51% (reported as the corrected total peak area of the sample). Two other interesting observations could be made: first, in several samples, MDMB‐INACA was the main SCRA present and second, in all but one of the samples (FL23/0367 being the exception) MDMB‐INACA was found alongside MDMB‐4en‐PINACA. Because MDMB‐INACA has not been found to be a thermal degradation product of MDMB‐4en‐PINACA [47], these findings support the suggestion that MDMB‐INACA may be a precursor for the synthesis of MDMB‐4en‐PINACA.

**TABLE 1:** Details of waxy‐ or putty‐like materials seized from Scottish prisons, divided in sample set 1 and 2, where the latter samples originated in sealed vape pods. Samples are arranged by seizure date. For security reasons, the prisons where the samples were seized are represented by a number rather than by name. Full analytical details are available in the Supporting Information (Table S3.1 and S4.1). CB_1_ activity was evaluated for the samples indicated in bold, using the in vitro CB_1_ bioassay.

	Sample ID	Prison	Seizure Date	Mass of Material (g)	Analysis Result	% corrected total peak area
Sample set 1 (*n* = 14)	FL23/0180	3	03/01/2023	4.4464	ADB‐BUTINACA	2.14
					MDMB‐4en‐PINACA	97.86
	FL23/0150	3	31/01/2023	2.8516	ADB‐BUTINACA	2.23
					MDMB‐4en‐PINACA	97.77
	FL23/0152	3	31/01/2023	0.7856	Δ^9^‐THC	—
	FL23/0167	3	06/03/2023	0.9088	Gabapentin	26.02
					Pregabalin	73.98
	**FL23/0174**	3	13/03/2023	5.2400	ADB‐BUTINACA	2.55
					MDMB‐4en‐PINACA	18.84
					MDMB‐INACA	78.61
	FL23/0134	11	03/04/2023	0.0170	Δ^9^‐THC	—
	**FL23/0135**	11	03/04/2023	1.1240	ADB‐BUTINACA	92.80
					MDMB‐4en‐PINACA	3.91
					MDMB‐INACA	3.30
	**FL23/0199**	3	11/04/2023	0.0352	ADB‐BUTINACA	5.62
					MDMB‐4en‐PINACA	12.87
					MDMB‐INACA	81.51
	**FL23/0188**	1	15/05/2023	0.6971	ADB‐BUTINACA	1.10
					MDMB‐4en‐PINACA	46.66
					MDMB‐INACA	52.24
	FL23/0445	11	24/07/2023	0.2045	ADB‐BUTINACA	13.74
					MDMB‐4en‐PINACA	10.69
					MDMB‐INACA	75.56
	FL23/0301	1	15/08/2023	0.5558	ADB‐BUTINACA	54.79
					MDMB‐4en‐PINACA	13.38
					MDMB‐INACA	31.83
	FL23/0302	1	15/08/2023	0.4886	ADB‐BUTINACA	15.64
					MDMB‐4en‐PINACA	14.06
					MDMB‐INACA	70.30
	FL23/0367	10	03/06/2023	0.8448	ADB‐BUTINACA	26.02
					MDMB‐INACA	73.98
	FL23/0460	1	12/10/2023	0.6082	MDMB‐4en‐PINACA	38.49
					MDMB‐INACA	61.51
Sample set 2 (vape pods; *n* = 8)	FL23/0229	3	26/03/2023	0.089	MDMB‐4en‐PINACA	—
	**FL23/0228**	3	28/03/2023	0.1789	MDMB‐4en‐PINACA	60.14
					MDMB‐INACA	39.86
	FL23/0232	3	30/03/2023	0.7426	MDMB‐4en‐PINACA	—
	**FL23/0290**	3	10/05/2023	0.5109	Δ^9^‐THC CBD CBN ADB‐BUTINACA^a^ MDMB‐4en‐PINACA^a^ MDMB‐INACA^a^	—
	**FL23/0293**	3	11/05/2023	0.1248	MDMB‐INACA	—
	**FL23/0291**	3	18/05/2023	0.0785	THCA	—
	FL23/0311	5	11/07/2023	0.1612	ADB‐BUTINACA	1.68
					MDMB‐4en‐PINACA	2.98
					MDMB‐INACA	2.49
					Bromazolam	92.85
	FL23/0422	7	08/08/2023	0.3091	MDMB‐4en‐PINACA	83.13
					MDMB‐INACA	16.87

^a^ These compounds were not identified during initial routine GC‐MS‐based screening, but, following a positive signal via the ‘untargeted’ activity‐based screening, were identified upon further analysis by LC‐QToF‐MS (see Figures S5.1–S5.3).

Of the remaining samples, two contained the phytocannabinoid Δ^9^‐THC, whereas one sample contained the anticonvulsant, analgesic, and anxiolytic medications pregabalin and gabapentin (73.98% and 26.02% of the corrected total peak area, respectively). The non‐prescription use of these gabapentinoids has already been reported in Scottish prisons as a standalone drug or in combination with other drugs [48]; however, this is the first report of their detection in waxy‐ or putty‐like materials.

**FIGURE 3:**
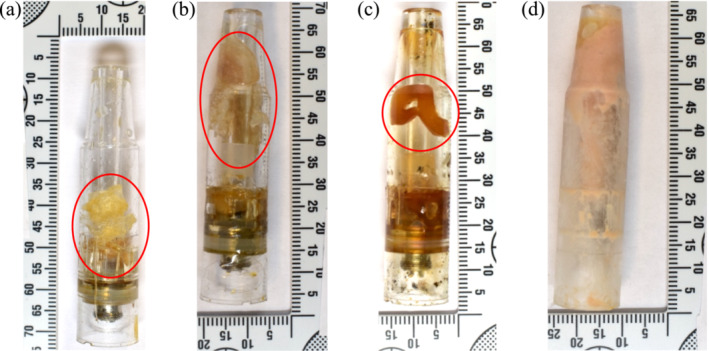
Examination photographs of samples with vape pods containing waxy‐ or putty‐like materials seized from the Scottish prisons: (a) FL23/0229, (b) FL23/0232, (c) FL23/0293, and (d) FL23/0311. The waxy‐ or putty‐like materials are circled in red apart for in (d) where the putty‐like material is throughout the whole vape pod.

In addition, between March 26 and August 8, 2023, eight samples seized from three Scottish prisons contained sealed vape pods with a piece of waxy‐ or putty‐like material inside. Details of the samples can be found in Table 1 (sample set 2), and full analytical details are available in the Supporting Information (Table S4.1). Example examination photographs of these materials can be found in Figure 3, and a complete set of photographs is available in the Supporting Information (Figure S7.4). The materials found in the vape pods weighed between 0.08 and 0.74 g (mean: 0.27 g, median: 0.17 g), demonstrating inconsistent dosing. It should be noted that for some samples, the material inside the vape pod was wrapped in cling film, making it impossible to separate the material and determine its exact weight. Cling film is used for the smuggling, storage, and likely distribution of these materials, but it is unclear why the cling film was also added to the vape pod. There is no evidence or intelligence to suggest drugs are being smuggled into the prisons inside the vape pods. Instead, drugs, including these waxy materials, papers, and powders/crushed tablets are added to the nicotine‐containing vape pods legally available for sale inside the prisons. This is supported by nicotine being detected in all eight samples found in a vape pod in this study (see Table S4.1).

SCRAs (MDMB‐4en‐PINACA, ADB‐BUTINACA, and/or MDMB‐INACA) were present in seven samples and one sample contained the phytocannabinoid tetrahydrocannabinolic acid (THCA). Interestingly, one sample (FL23/0293) only contained MDMB‐INACA, whereas it was detected alongside MDMB‐4en‐PINACA in four samples. In two samples, the SCRAs were detected alongside other drugs. Sample FL23/0290 was initially thought to contain only phytocannabinoids (Δ^9^‐THC, cannabidiol (CBD), and cannabinol (CBN)), but follow‐up analysis indicated the presence of SCRAs, as discussed further in Section 3.3. Sample FL23/0311 had bromazolam as its main constituent, with the SCRAs possibly present as the result of cross‐contamination (e.g., owing to prior use of the vape pod). MeOH blanks were run on the GC‐MS prior to each sample, so this cross‐contamination was not the result of the sample preparation or instrumental analysis. The detection of a benzodiazepine is not unexpected given that novel benzodiazepines are among the most commonly detected substances in Scottish prisons. Benzodiazepine‐infused papers have previously been detected in vape pods, although it is thought that they are unintentionally vaped due to the belief that they contain SCRAs [27].

Additionally, between March 6 and August 29, 2023, 11 samples containing waxy‐ or putty‐like material wrapped in cling film were seized from an English prison after they were thrown over the prison boundary. Details and examination photographs of these samples can be found in the Supporting Information (Table S6.1, Figure S7.5). Interestingly, these samples were much greater in weight than those seized from the Scottish prisons, ranging from 7.47 to 27.13 g (mean: 19.25 g; median: 21.42 g). All the samples were crumbly and slightly oily in texture (Figure 2i), and upon boiling in water, they appeared as an oily, hydrophobic liquid. Also, for these samples, MDMB‐INACA was found in proportions up to 35% in combination with MDMB‐4en‐PINACA based on quantitative NMR analysis. Additionally, 4F‐MDMB‐BUTINACA was identified by LC‐QToF‐MS in two samples, although it was not present at concentrations detectable by NMR. Finally, dimethylformamide (DMF) was identified by NMR in all 11 samples. DMF is a common solvent used in the synthesis of SCRAs [49, 50, 51] and is also included as part of the “DIY kits” for the synthesis of SCRAs from precursors, as can be seen in the screenshots provided in the Supporting Information (Figures S1.1 and S2). Therefore, the DMF was likely present as an impurity from the synthetic method.

Overall, the waxy‐ or putty‐like materials were reminiscent of BHO materials, which also appear in different forms, depending on the manufacturing process and environmental conditions, ranging from soft wax called “budder” and “earwax,” to hard and translucent, referred to as “shatter” [34, 35]. Typically, BHO materials are prepared by liquid butane extraction of herbal cannabis, followed by evaporation of the butane from the resulting solution [36, 52]. However, other solvents, like propane, or alternative methods, such as carbon dioxide extraction, have also been reported [53, 54]. Given the resemblance of these waxy‐ or putty‐like materials, they may follow a similar production process, but apart from the drugs, the other components of the materials were insoluble in organic solvent and therefore unable to be determined. This should be explored more in the future to help inform how these materials are being produced and the potential harms from the production and use of this sample matrix.

In the Scottish prisons, vaping appears to be the preferred method of using waxy‐ or putty‐like materials, as evidenced by their presence in vape pods. Besides their forms, their mode of consumption is also reminiscent of BHO materials, which are commonly vaped as e‐cigarettes or other electronic vaporizers, as well as dabbed and heated on a nail to vaporize [31, 32, 52, 55]. The vaping and dabbing of BHO materials has been found to cause severe adverse effects on the respiratory system [36, 55, 56, 57]; therefore, the vaping of these waxy‐ or putty‐like materials may contribute to adverse effects. Besides vaping, these waxy‐ or putty‐like materials may also be used via boiling in kettles, a method previously observed in Scottish prisons for drug‐infused materials, followed by ingestion of the resulting liquid [27]. In the English and Welsh prisons, it has been suggested that these putty samples are potentially being dissolved into a carrier liquid and then used to infuse papers within the prisons. Pieces of infused paper can then be inserted into a vape to be vaporized, which is consistent with seized vape cartridges being found with paper scraps inside [23].

### 3.2 Determination of In Vitro CB_1_ and CB_2_ Receptor Activity

**FIGURE 4:**
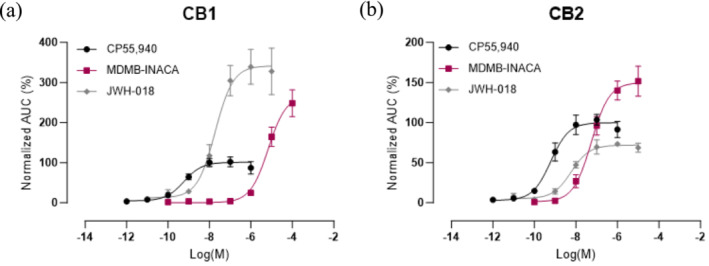
Concentration–response curves at (a) CB_1_ and (b) CB_2_ of MDMB‐INACA, the reference standard CP55,940 and JWH‐018. Each datapoint represents the mean ± SEM of 3 or more independent experiments. All data were normalized to the maximal response of CP55,940. Note the difference in scale for CB_1_ and CB_2_.

Given the fact that the relatively new tail‐less SCRA MDMB‐INACA was found in a large number of the investigated samples seized in prisons and no information was available on its pharmacological characteristics, its intrinsic cannabinoid receptor activation potential was assessed. This was done using an in vitro cell‐based assay in which receptor activation is monitored via the functional complementation of a split nanoluciferase enzyme. In short, activation of CB_1_ or CB_2_, which are each linked to an inactive subunit of the enzyme, results in recruitment of the intracellular signaling protein βarr2, which is fused to the other, complementary subunit. This event brings the two subunits in close proximity, leading to the restoration of the enzymatic activity and generation of bioluminescence. The concentration–response curves obtained for MDMB‐INACA, along with those for the reference standards CP55,940 and JWH‐018, are shown in Figure 4.

The corresponding potency (*EC*_50_) and (relative) efficacy (*E*_max_) values are shown in Table 2. CP55,940, which was used for normalization and for which the *E*_max_ was arbitrarily set at 100%, had a potency of 0.58 and 0.61 nM at CB_1_ and CB_2_, respectively, which is in line with earlier published work [42]. Based on the concentration–response curves, MDMB‐INACA had a potency at CB_1_ that was approximately 10,000 times lower than the reference compound CP55,940, with an efficacy of 268%. As a comparison, JWH‐018, a prototypical SCRA, combined a high potency at CB_1_ (18.8 nM) with a slightly higher efficacy (342%), similar to previously reported values for this SCRA [42]. On the other hand, at CB_2_, MDMB‐INACA had the highest efficacy of the investigated compounds (150%, relative to CP55,940), exceeding that of JWH‐018 (71.9%), but it remained the least potent compound of the panel, with an *EC*_50_ of 55.2 nM, which is about 10 and 100 times higher than that of JWH‐018 (5.90 nM) and CP55,940 (0.61 nM), respectively. This profile at both receptors closely resembles that of other tail‐less SCRAs, including ADB‐INACA and the brominated analogs ADB‐5′Br‐INACA and MDMB‐5′Br‐INACA [14]. This demonstrates that despite the absence of a tail structure, tail‐less SCRAs maintain some potential to activate the cannabinoid receptors, albeit at a low potency. The observed high efficacies at CB_1_, compared with those reported in the literature (obtained via other assays), are consistent with earlier observations and can be related to assay‐specific differences [4, 43]. For example, in assays measuring a more downstream event, a plateau can be reached owing to signal amplification, thereby resulting in *E*_max_ values converging at 100% (also referred to as the “ceiling effect”) [4].

**TABLE 2:** Potency (*EC*_50_) and efficacy (*E*_max_) values of MDMB‐INACA and JWH‐018, relative to the reference standard CP55,940 at the CB_1_ and CB_2_ receptor obtained using NanoBiT® βarr2 recruitment bioassays.

	CB_1_		CB_2_	
	*EC*_50_ (nM)	*E*_max_ (%)	*EC*_50_ (nM)	*E*_max_ (%)
MDMB‐INACA	6.81 × 10^3^ (3.94 × 10^3^–11.4 × 10^3^)	268 (233–305)	55.2 (25.9–111)	150 (134–167)
JWH‐018	18.8 (8.20–44.4)	342 (300–385)	5.90 (2.94–11.5)	71.9 (66–77.9)
CP55,940	0.58 (0.27–1.17)	101 (91.1–112)	0.61 (0.29–1.21)	99.8 (90.3–110)

### 3.3 Evaluation of the Intrinsic CB_1_ Receptor Activation Potential of Waxy‐ and Putty‐Like Samples

**FIGURE 5:**
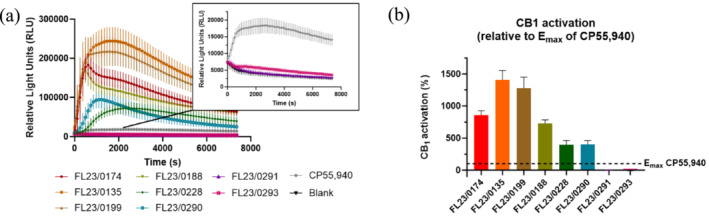
(a) CB_1_ activation profiles obtained for extracts from eight waxy‐ or putty‐like samples alongside CP55,940 (at the concentration that resulted in the highest signal) and solvent control, with a zoom‐in of the activation profile from FL23/0291 and FL23/0293. (b) CB_1_ activity from the corresponding waxy‐or putty‐like samples compared with the efficacy (*E*_max_) of CP55,940. Data are presented as relative light units (RLU) ± SEM from three independent experiments.

The CB_1_‐βarr2 recruitment assay was also used to evaluate the in vitro CB_1_ activation by eight seized samples of waxy‐ or putty‐like material (Figure 5a). The eight samples (as indicated in bold in Table 1) were selected to create a subset of the materials from the Scottish prisons. The samples were divided between sample set 1 and 2 (Table 1). As GC‐MS analysis had already been performed to identify the contents, samples were chosen to incorporate the variety of cannabinoids detected in the materials, including phytocannabinoids and SCRAs. This activity‐based assay has previously been used to gain insight into the extent of CB_1_ activation by biological matrices such as urine, blood, plasma, or serum [45, 46] and by (laced) herbal materials [58] and powders [14, 42, 59]. The obtained activity data allow the preparations to be distinguished or characterized based on their inherent, combined CB_1_ activity, being determined by both the identity and concentration of the SCRAs. This CB_1_ activity can be translated to the “strength” of a preparation, serving as an indicator of its potential cannabinoid‐related harm. As an illustration, comparison to the efficacy (*E*_max_) of CP55,940 (Figure 5b) revealed that FL23/0135 and FL23/0199, containing ADB‐BUTINACA, MDMB‐4en‐PINACA, and MDMB‐INACA, exhibited the highest CB_1_ activity, which was about 14 and 12.5 times higher than the *E*_max_ of CP55,940 and JWH‐018, respectively. Also, FL23/0174, FL23/0188, FL23/0228, and FL23/0290, all containing MDMB‐4en‐PINACA in combination with ADB‐BUTINACA and/or MDMB‐INACA, yielded a clear CB_1_ activation, being approximately four to eight times higher than the *E*_max_ of CP55,940. In other words, the pronounced CB_1_ activation by these preparations is suggestive of a significant “strength,” which could be expected given the highly efficacious and potent nature of the SCRAs (i.e., ADB‐BUTINACA, MDMB‐4en‐PINACA) found in these samples. Earlier in vitro data on MDMB‐4en‐PINACA and ADB‐BUTINACA revealed a high CB_1_ activation potential with *E*_max_ values of 679% and 728%, normalized to CP55,940, respectively, and potencies in the low nanomolar range (*EC*_50_ = 1.88 and 19 nM, respectively) [4, 60]. Despite these seized samples containing different compositions of SCRAs, the presence of (even a trace amount of) a highly potent SCRA has the ability to define the CB_1_ profile. For example, FL23/0199 resulted in a strong CB_1_ activation even though MDMB‐INACA was the most abundant compound (81.5% of total peak area).

As anticipated, sample FL23/0291 did not yield any CB_1_ activity, as there were no SCRAs present in this sample. An extract from sample FL23/0293, containing MDMB‐INACA, only activated CB_1_ to a limited extent, as the profile was clearly below that obtained for CP55,940, though still distinguishable from the solvent control (Figure 5a, zoom‐in). This was expected as MDMB‐INACA had a very low potency at CB_1_, being more than 10,000 times lower than CP55,940. This implies that most likely relatively high, possibly not attainable, concentrations would be required to yield a strong CB_1_ activation.

Of special note is sample FL23/0290. Initially, only Δ^9^‐THC, CBD, CBN, and nicotine had been identified in this sample via GC‐MS; however, an extract of this sample yielded a clear CB_1_ activation uncharacteristic of these identified phytocannabinoids. This was indicative of the presence of one or more SCRAs because Δ^9^‐THC (if extracted at all) is only expected to yield a very limited CB_1_ activation, as it is only a weak partial agonist at CB_1_ (i.e., <10% efficacy compared with that of CP55,940) in the employed assay [44, 61]. Moreover, CP55,940 has a moderate efficacy at CB_1_ relative to other SCRAs, for example, ADB‐BUTINACA (*E*_max_ = 728%, normalized to CP55,940) and MDMB‐4en‐PINACA (*E*_max_ = 679%, normalized to CP55,940) [4, 60]. Consequently, compared with CP55,940 and other SCRAs, it was unlikely that the extracted Δ^9^‐THC would substantially contribute to an increase in signal in the bioassay. Further investigation via LC‐QToF‐MS confirmed the presence of MDMB‐4en‐PINACA, ADB‐BUTINACA, and MDMB‐INACA, which were also later found in the initial GC‐MS data through extraction of the ion chromatograms. The detection of relatively low concentrations further illustrates the high sensitivity and the useful application of the in vitro bioassay in the context of a first‐line screening tool. It may complement current analytical techniques by flagging samples for which the presence of SCRAs is suspected and allowing a more efficient further analytical evaluation.

Although the in vitro assay provides valuable insights into the total “strength” of a preparation in terms of CB_1_ activity, a direct translation of the in vitro CB_1_ activation to the in vivo situation is hampered by different aspects, such as the method and intensity of use, as well as the SCRA's bioavailability, metabolism, and blood–brain‐barrier permeability. Nevertheless, when a preparation yields no or a relatively low CB_1_ activation (e.g., samples FL23/0291 and FL23/0293), it can be cautiously rationalized that the “strength” (and hence the potential to result in CB_1_‐linked toxicity) of these preparations is anticipated to be lower than that of preparations that yield high CB_1_ activation. Therefore, activity‐based analysis may also serve a purpose in the context of harm reduction by identifying those preparations that are likely to pose a higher risk.

## 4 Conclusion

In this study, waxy‐ or putty‐like materials were reported for the first time as a novel drug preparation for SCRAs and other drugs seized from Scottish and English prisons. Additionally, the tail‐less SCRA MDMB‐INACA was detected for the first time in samples seized from Scottish prisons. A total of 22 of these new waxy‐ or putty‐like materials were seized from Scottish prisons in 2023, with eight found in sealed vape pods. The seized samples were found positive for SCRAs (MDMB‐4en‐PINACA, MDMB‐INACA, and/or ADB‐BUTINACA), phytocannabinoids, novel benzodiazepines, and/or gabapentinoids. In addition, 11 waxy‐ or putty‐like materials were seized from an English prison in 2023. Most of these contained the combination of MDMB‐4en‐PINACA alongside MDMB‐INACA, further supporting the precursor rationale. The latter was also pharmacologically characterized, using in vitro CB_1_ and CB_2_ βarr2 recruitment assays, revealing that MDMB‐INACA had a moderate efficacy (*E*_max_ = 268% relative to CP55,940) but a low potency at CB_1_ (*EC*_50_ = 6.81 × 10^3^ nM). In addition, to aid the detection of SCRAs in these new matrices, the in vitro intrinsic CB_1_ activation potential of eight waxy‐ or putty‐like materials was assessed. Six of these samples (all containing MDMB‐4en‐PINACA alongside ADB‐BUTINACA and/or MDMB‐INACA) showed high CB_1_ activity, whereas the sample that lacked SCRAs or only contained the SCRA MDMB‐INACA showed no or weak activity at CB_1_, respectively. Furthermore, the application of the in vitro CB_1_ bioassay was able to reveal the presence of SCRAs in a waxy sample that had initially been labeled as SCRA‐negative based on GC‐MS analysis, but upon follow‐up, LC‐QToF‐MS analysis was confirmed to be SCRA‐positive. This finding underscores the effectiveness of the bioassay for detecting SCRAs in waxy‐ or putty‐like materials.

## Conflicts of Interest

The authors declare no conflicts of interest.

## Supporting information


**Data S1** Supporting Information

## Data Availability

All data supporting the findings of this study are available within the paper and its Supporting Information.
