# Supplementary material for: Waxy‐ or Putty‐Like Materials as a Novel Drug Preparation for Synthetic Cannabinoid Receptor Agonists: Detection in Prisons and In Vitro Cannabinoid Receptor Activity
Source: Drug Test Anal. 2024 Oct 15;17(7):1118–30. doi: 10.1002/dta.3817 (PMC12209699; doi:10.1002/dta.3817)
Supplement: Supplementary file 1 — Data S1 Supporting Information [file DTA-17-1118-s001.docx]

**Waxy- or putty-like materials as a novel drug preparation for synthetic cannabinoid receptor agonists: Detection in prisons and *in vitro* cannabinoid receptor activity**

Axelle Timmerman^1^, Marie H. Deventer^1^, Rachael Andrews^2^, Robert Reid^3^, Victoria Marland^3^, Darren Edwards^4^, Christopher Pudney^2,5^, Niamh Nic Daéid^3^, Christophe P. Stove^1^, Caitlyn Norman^3,6,*^

^1^Laboratory of Toxicology, Department of Bioanalysis, Faculty of Pharmaceutical Sciences, Ghent University, Ghent, Belgium

^2^Department of Life Sciences, University of Bath, Bath, UK

^3^Leverhulme Research Centre for Forensic Science, School of Science and Engineering, University of Dundee, Dundee, UK

^4^Drug Discovery Unit, School of Life Sciences, University of Dundee, Dundee, UK

^5^Centre for Bioengineering and Biomedical Technologies, University of Bath, Bath, UK

^6^Department of Biomedical and Clinical Science, Division of Clinical Chemistry and Pharmacology, Linköping University, Linköping, Sweden

^*^Corresponding Author: caitlyn.norman@liu.se

**Supplementary Information**

**Section 1:** Screenshots from websites selling the synthetic cannabinoid “DIY kits”.

**Section 2:** Correction factors for EI-MS detector response of compounds detected in Scottish Prisons.

**Section 3:** Full analytical data for samples of waxy- or putty-like materials seized in the Scottish prisons.

**Section 4:** Full analytical data for samples with e-cigarettes containing waxy- or putty-like materials seized in the Scottish prisons.

**Section 5:** LC-QToF-MS analytical data for sample FL23/0290.

**Section 6:** Full analytical data for samples of waxy- or putty-like material seized in English prisons.

**Section 7:** Examination photos of all samples of waxy- or putty-like materials seized in the Scottish and English prisons.

**Section 1: Screenshots from websites selling the synthetic cannabinoid “DIY kits”.**


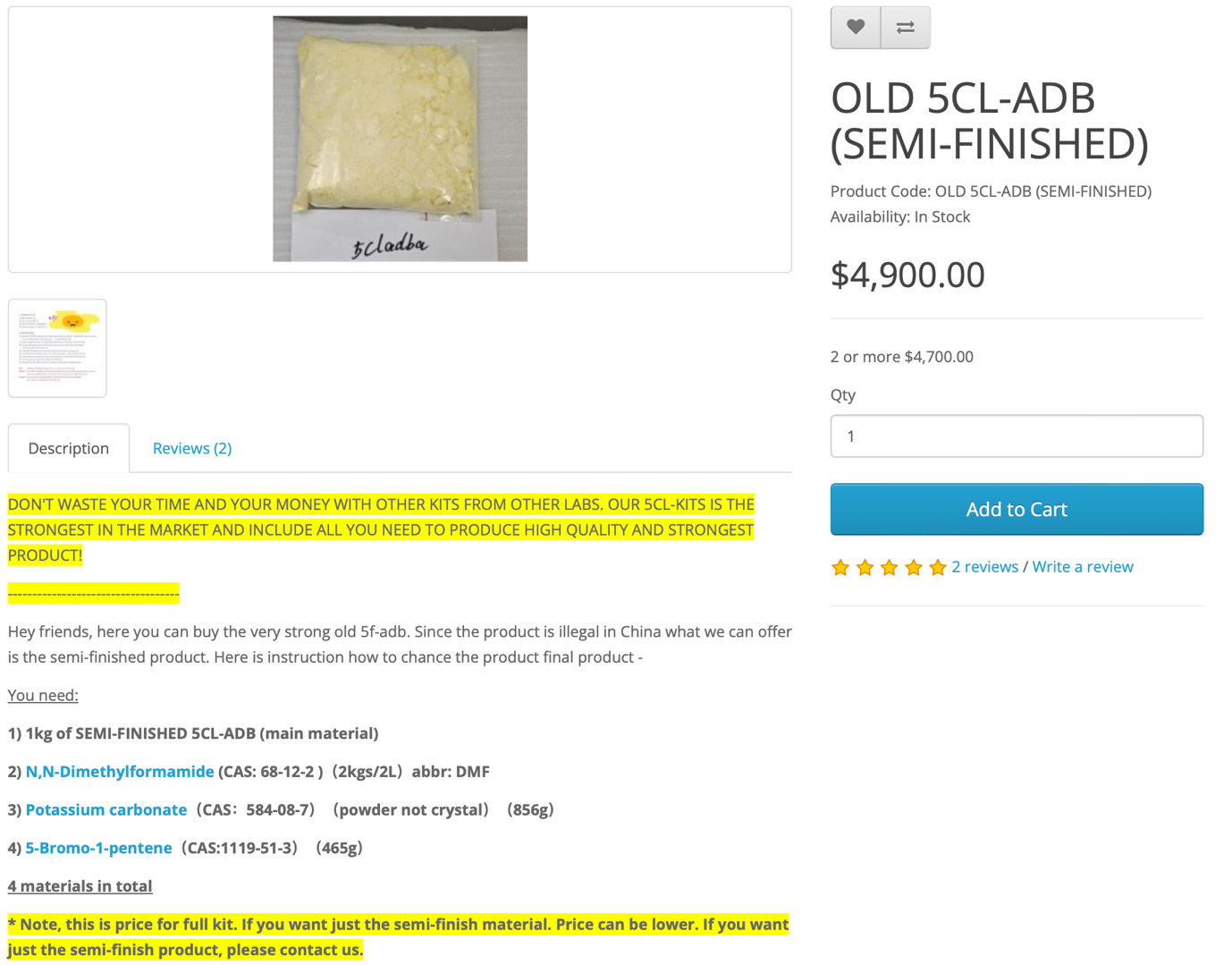


Figure S1.1: Screenshot from website selling DIY kits for synthetic cannabinoids.


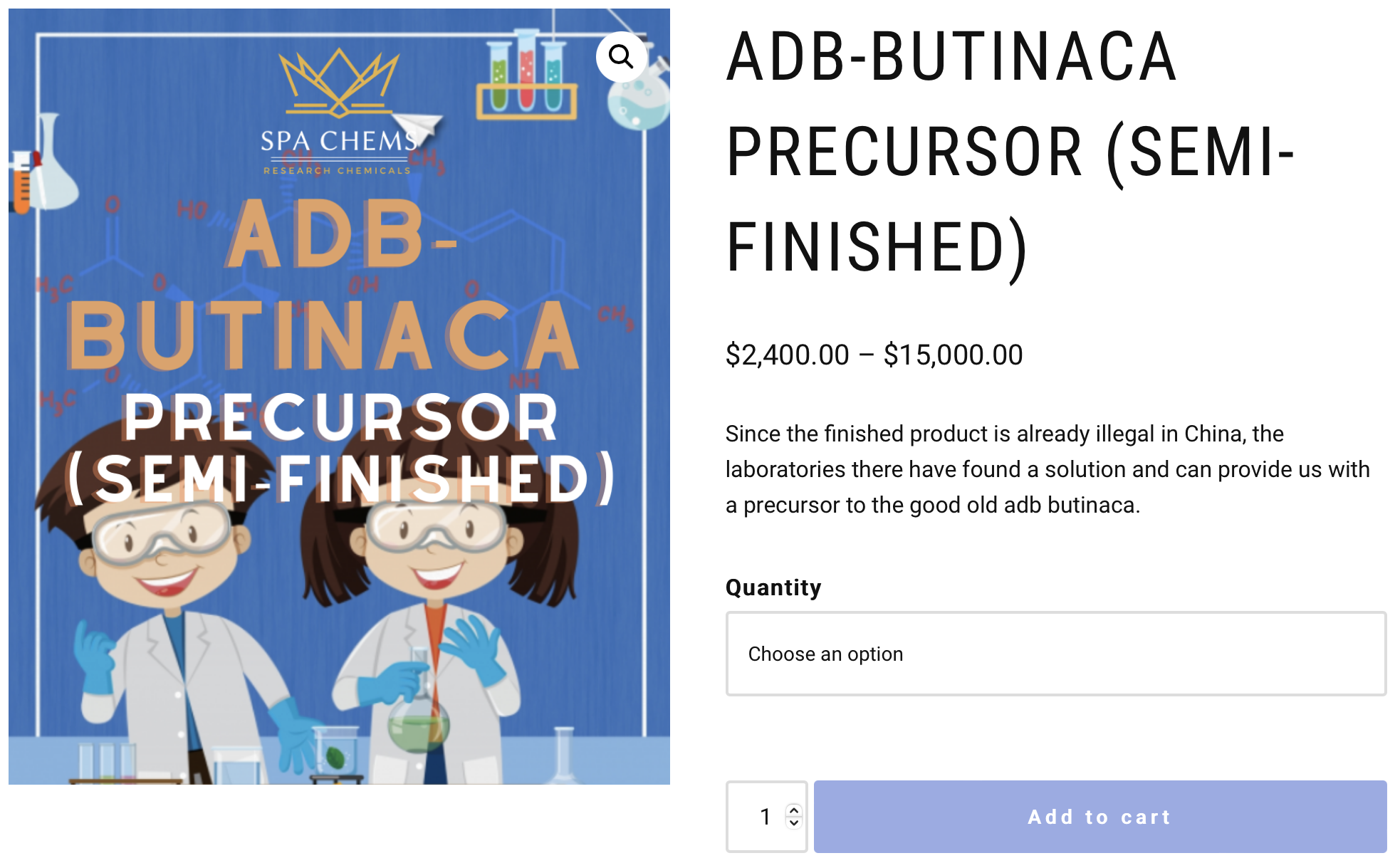


 
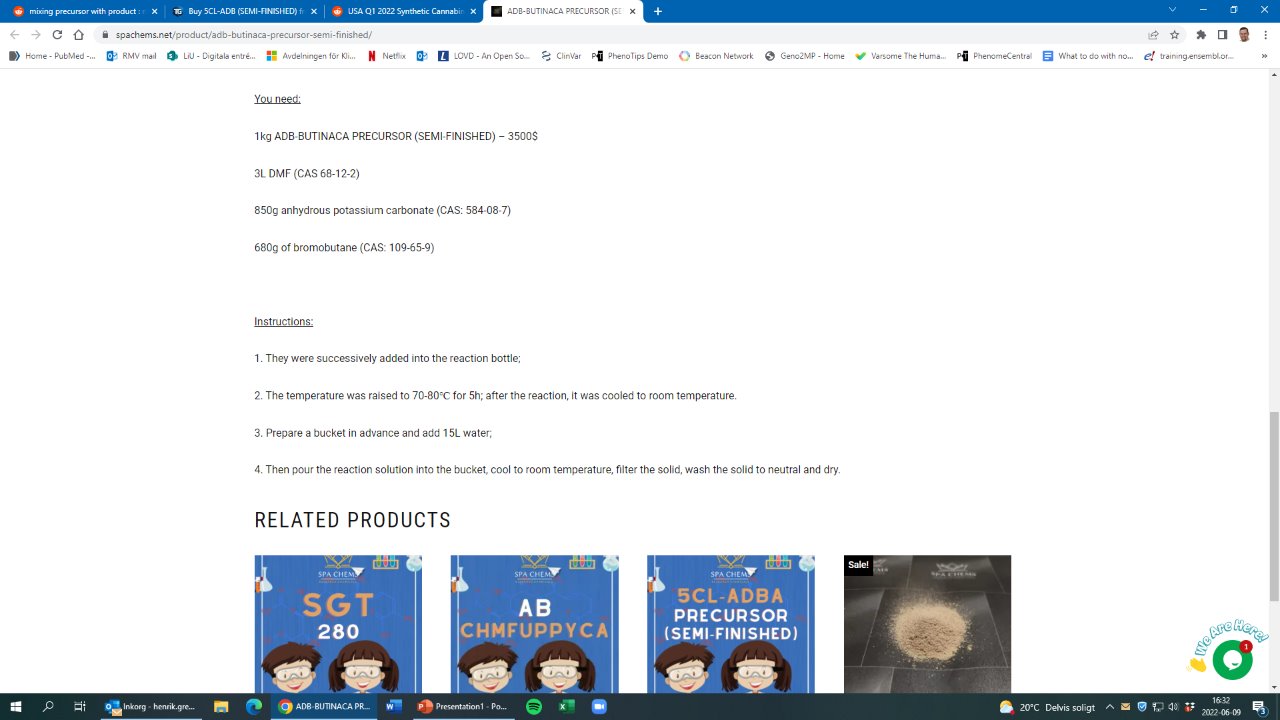


Figure S1.2: Screenshot from website selling DIY kits for synthetic cannabinoids (website no longer active).

**Section 2: Correction factors for EI-MS detector response of compounds detected in Scottish Prisons.**

For samples with a mixture of compounds, the percentage total peak area for each compound was determined by comparing the peak areas of each compound to the total peak area of all active components in the sample. The percentage peak area was then corrected to account for the different EI-MS detector response of each compound by running the samples on the GC-MS alongside a mixture prepared from reference materials at the same concentrations (100 μg/mL). Based on comparison of the peak areas of the reference materials, correction factors were calculated and applied to the peak areas of each compound in a sample. The correction factors applied for each compound can be found in Table S2.1.

Table S2.1: Correction factors for EI-MS detector response applied to compounds detected in Scottish Prisons for calculating percentage of total peak area. For all samples containing MDMB-INACA, MDMB-INACA was set to the value of 1 and corrections were applied to the other compounds. For all other samples, MDMB-4en-PINACA was set to the value of 1 and corrections were applied to the other compounds.

| **Corrected compounds** | **% of total** | **Compounds set to value of 1** | |
| --- | --- | --- | --- |
|  |  | MDMB-INACA | MDMB-4en-PINACA |
| MDMB-INACA | 18.5 | - | - |
| MDMB-4en-PINACA | 39.4 | 0.469 | - |
| ADB-BUTINACA | 27.0 | 0.684 | 1.459 |
| Bromazolam | 15.2 | 1.218 | 2.597 |

**Section 3: Full analytical data for samples of waxy- or putty-like materials seized in the Scottish prisons.**

Table S3.1: Full analytical data for samples of waxy- or putty-like materials seized in the Scottish prisons. Samples are arranged by seizure date. For security reasons, the prison where the sample was seized is represented by a number rather than the name of the establishment. Details of the GC-MS analysis are provided, including retention time (RT) of the compound, RT of the reference standard (ref std), MS library R-match (x/1000), and the mass (m/z) of the compound. For some samples, the details of the confirmatory analysis by UPLC-QToF-MS are provided, including exact mass ([M+H]^+^ (amu)), theoretical monoisotopic mass (amu), mass error (ppm), and MS/MS fragments (amu).

| **Sample ID** | **Establishment** | **Seizure Date** | **Mass of Material (g)** | **GC-MS** | | | | | **UPLC-QToF-MS/MS** | | | | |
| --- | --- | --- | --- | --- | --- | --- | --- | --- | --- | --- | --- | --- | --- |
|  |  |  |  | **Analysis Result** | **RT (mins)** | **R-Match (x/1000)** | **RT ref std (mins)** | **m/z (amu)** | **λmax (nm)** | **[M+H]^+^ (amu)** | **Theoretical Mass (amu)** | **Mass Error (ppm)** | **MS/MS Fragments (amu)** |
|  |  |  |  |  |  |  |  |  |  |  |  |  |  |
| FL23/0180 | 3 | 03/01/2023 | 4.4464 | ADB-BUTINACA | 9.204 | 906 | 9.209 | 330 |  |  |  |  |  |
|  |  |  |  | MDMB-4en-PINACA | 8.940 | 939 | 8.906 | 357 |  |  |  |  |  |
| FL23/0150 | 3 | 31/01/2023 | 2.8516 | ADB-BUTINACA | 9.221 | 909 | 9.209 | 330 |  |  |  |  |  |
|  |  |  |  | MDMB-4en-PINACA | 8.940 | 936 | 8.906 | 357 |  |  |  |  |  |
| FL23/0152 | 3 | 31/01/2023 | 0.7856 | Δ^9^-THC | 8.723 | 969 |  | 314 |  |  |  |  |  |
| FL23/0167 | 3 | 06/03/2023 | 0.9088 | Gabapentin | 4.217 | 922 | 4.151 | 171 |  |  |  |  |  |
|  |  |  |  | Pregabalin | 3.247 | 947 | 3.299 | 159 |  |  |  |  |  |
| FL23/0174 | 3 | 13/03/2023 | 5.2400 | ADB-BUTINACA | 9.318 | 881 | 9.204 | 330 | 303 | 331.2128 | 331.2134 | -1.8 |  |
|  |  |  |  | MDMB-4en-PINACA | 8.969 | 941 | 8.900 | 357 | 303 | 358.2110 | 358.2131 | -5.9 |  |
|  |  |  |  | MDMB-INACA | 8.746 | 891 | 8.666 | 289 | 303 | 290.1513 | 290.1505 | 2.8 | 86.0981, 145.0413, 162.0644, 230.1162 |
| FL23/0134 | 11 | 03/04/2023 | 0.0170 | Δ^9^-THC | 8.717 | 968 |  | 314 |  |  |  |  |  |
| FL23/0135 | 11 | 03/04/2023 | 1.1240 | ADB-BUTINACA | 9.215 | 875 | 9.209 | 330 |  |  |  |  |  |
|  |  |  |  | MDMB-4en-PINACA | 8.900 | 926 | 8.906 | 357 | 303 | 358.2127 | 358.2131 | -1.1 |  |
|  |  |  |  | MDMB-INACA | 8.717 | 882 | 8.666 | 289 | 303 | 290.1508 | 290.1505 | 1 | 86.1002, 145.0401, 162.0647, 230.1300 |
| FL23/0199 | 3 | 11/04/2023 | 0.0352 | ADB-BUTINACA | 9.247 | 890 | 9.193 | 330 |  |  |  |  |  |
|  |  |  |  | MDMB-4en-PINACA | 8.921 | 937 | 8.900 | 357 |  |  |  |  |  |
|  |  |  |  | MDMB-INACA | 8.717 | 876 | 8.666 | 289 |  |  |  |  |  |
| FL23/0188 | 1 | 15/05/2023 | 0.6971 | ADB-BUTINACA | 9.267 | 885 | 9.204 | 330 | 303 | 331.2186 | 331.2174 | 3.6 |  |
|  |  |  |  | MDMB-4en-PINACA | 8.969 | 937 | 8.900 | 357 | 303 | 358.2130 | 358.2131 | -0.3 |  |
|  |  |  |  | MDMB-INACA | 8.746 | 879 | 8.666 | 289 | 303 | 290.1519 | 290.1505 | 4.8 | 86.0976, 145.0406 |
| FL23/0445 | 11 | 24/07/2023 | 0.2045 | ADB-BUTINACA | 9.056 | 827 | 9.026 | 330 |  |  |  |  |  |
|  |  |  |  | MDMB-4en-PINACA | 8.765 | 943 | 8.752 | 357 |  |  |  |  |  |
|  |  |  |  | MDMB-INACA | 8.575 | 884 | 8.532 | 289 |  |  |  |  |  |
| FL23/0301 | 1 | 15/08/2023 | 0.5558 | ADB-BUTINACA | 9.229 | 886 | 9.142 | 330 |  |  |  |  |  |
|  |  |  |  | MDMB-4en-PINACA | 8.872 | 939 | 8.849 | 357 |  |  |  |  |  |
|  |  |  |  | MDMB-INACA | 8.675 | 863 | 8.618 | 289 |  |  |  |  |  |
| FL23/0302 | 1 | 15/08/2023 | 0.4886 | ADB-BUTINACA | 9.191 | 860 | 9.142 | 330 |  |  |  |  |  |
|  |  |  |  | MDMB-4en-PINACA | 8.868 | 938 | 8.849 | 357 |  |  |  |  |  |
|  |  |  |  | MDMB-INACA | 8.686 | 872 | 8.618 | 289 |  |  |  |  |  |
| FL23/0367 | 10 | 03/06/2023 | 0.8448 | ADB-BUTINACA | 9.108 | 870 | 9.139 | 289 |  |  |  |  |  |
|  |  |  |  | MDMB-INACA | 8.601 | 853 | 8.601 | 289 |  |  |  |  |  |
| FL23/0460 | 1 | 12/10/2023 | 0.6082 | MDMB-4en-PINACA | 8.779 | 939 | 8.757 | 330 |  |  |  |  |  |
|  |  |  |  | MDMB-INACA | 8.605 | 870 | 8.537 | 357 |  |  |  |  |  |

**Section 4: Full analytical data for samples with e-cigarettes containing waxy- or putty-like materials seized in the Scottish prisons.**

Table S4.1: Full analytical data for samples with e-cigarettes containing waxy- or putty-like materials seized in the Scottish prisons. Samples are arranged by seizure date. For security reasons, the prison where the sample was seized is represented by a number rather than the name of the establishment. Details of the GC-MS analysis are provided, including retention time (RT) of the compound, RT of the reference standard (ref std), MS library R-match (x/1000), and the mass (m/z) of the compound.

| **Sample ID** | **Establishment** | **Seizure Date** | **Mass of Material (g)** | **Analysis Result** | **RT (mins)** | **R-Match (x/1000)** | **RT ref std (mins)** | **m/z (amu)** |  |
| --- | --- | --- | --- | --- | --- | --- | --- | --- | --- |
| FL23/0229-4 | 3 | 26/03/2023 | 0.0890 | MDMB-4en-PINACA | 8.889 | 912 | 8.900 | 357 |  |
|  |  |  |  | Nicotine | 5.513 | 956 | - | 162 |  |
| FL23/0228-1 | 3 | 28/03/2023 | 0.1789 | MDMB-4en-PINACA | 8.895 | 910 | 8.900 | 357 |  |
|  |  |  |  | MDMB-INACA | 8.654 | 891 | 8.666 | 289 |  |
|  |  |  |  | Nicotine | 5.513 | 954 | - | 162 |  |
| FL23/0232 | 3 | 30/03/2023 | 0.7426 | MDMB-4en-PINACA | 8.895 | 934 | 8.900 | 357 |  |
|  |  |  |  | Nicotine | 5.513 | 959 | - | 162 |  |
| FL23/0290 | 3 | 10/05/2023 | 0.5109 | Δ^9^-THC | 7.148 | 968 | - | 314 |  |
|  |  |  |  | Cannabidiol | 6.827 | 940 | - | 314 |  |
|  |  |  |  | Cannabinol | 7.365 | 962 | - | 310 |  |
|  |  |  |  | ADB-BUTINACA* | - | - | - | 330 |  |
|  |  |  |  | MDMB-4en-PINACA* | 8.750 | 869 | 8.752 | 357 |  |
|  |  |  |  | MDMB-INACA* | 8.530 | 854 | 8.534 | 289 |  |
|  |  |  |  | Nicotine | 5.472 | 955 | - | 162 |  |
| FL23/0293 | 3 | 11/05/2023 | 0.1248 | MDMB-INACA | 8.620 | 874 | 8.637 | 289 |  |
|  |  |  |  | Nicotine | 5.484 | 932 | - | 162 |  |
| FL23/0291 | 3 | 18/05/2023 | 0.0785 | Tetrahydrocannabinolic acid (THCA) | 8.663 | 932 | - | 358 |  |
|  |  |  |  | Nicotine | 5.472 | 948 | - | 162 |  |
| FL23/0311 | 5 | 11/07/2023 | 0.1612 | ADB-BUTINACA | 9.101 | 902 | 9.132 | 330 |  |
|  |  |  |  | MDMB-4en-PINACA | 8.818 | 925 | 8.836 | 357 |  |
|  |  |  |  | MDMB-INACA | 8.589 | 876 | 8.592 | 289 |  |
|  |  |  |  | Bromazolam | 10.646 | 973 | 10.645 | 352 |  |
|  |  |  |  | Nicotine | 5.442 | 954 | - | 162 |  |
| FL23/0422 | 7 | 08/08/2023 | 0.3091 | MDMB-4en-PINACA | 8.772 | 935 | 8.757 | 357 |  |
|  |  |  |  | MDMB-INACA | 8.605 | 874 | 8.537 | 289 |  |
|  |  |  |  | Nicotine | 5.370 | 947 | - | 162 |  |

* MDMB-4en-PINACA and MDMB-INACA were not identified in FL23/0290 during the routine screening but were found after extracting the characteristic ions (m/z 145, 213, 301, 357 for MDMB-4en-PINACA and m/z 145, 201, 233, 289 for MDMB-INACA). There were also characteristic ions of ADB-BUTINACA present but the peaks were too small to obtain any mass spectra; however, its presence was confirmed by LC-QToF-MS as shown in Section 5.

**Section 5: LC-QToF-MS analytical data for sample FL23/0290.**


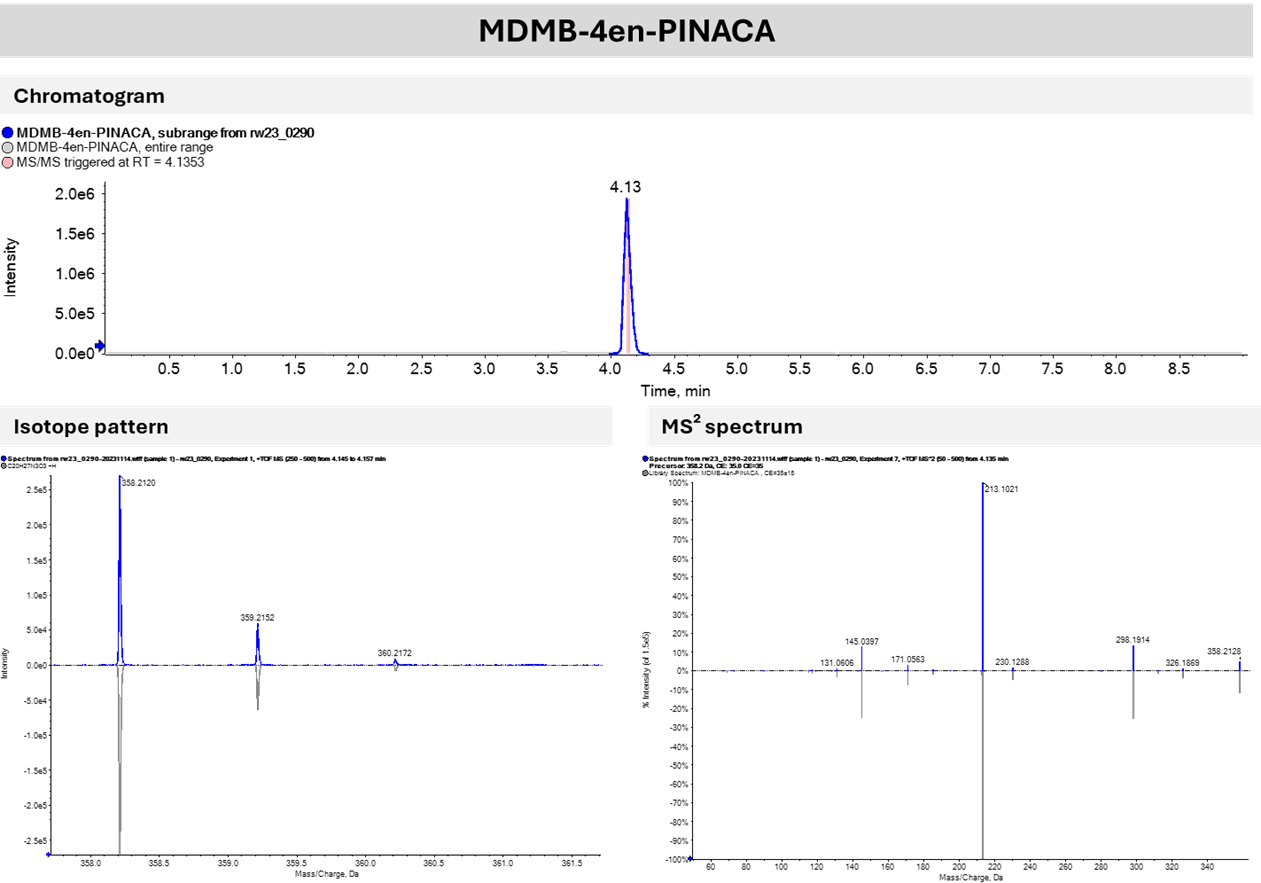


Figure S5.1: Identification of MDMB-4en-PINACA in sample FL23/0290 by LC-QToF-MS.


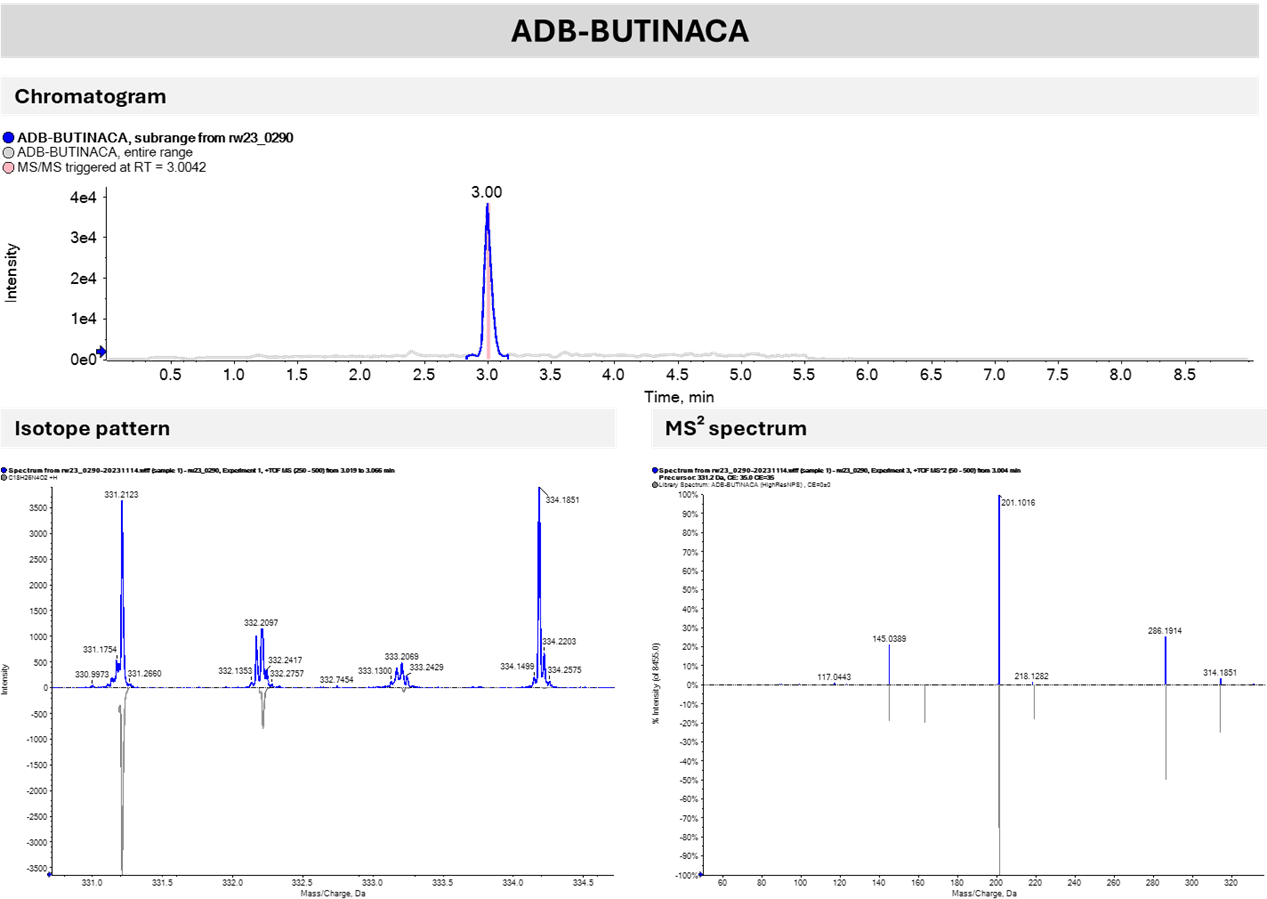


Figure S5.2: Identification of ADB-BUTINACA in sample FL23/0290 by LC-QToF-MS.


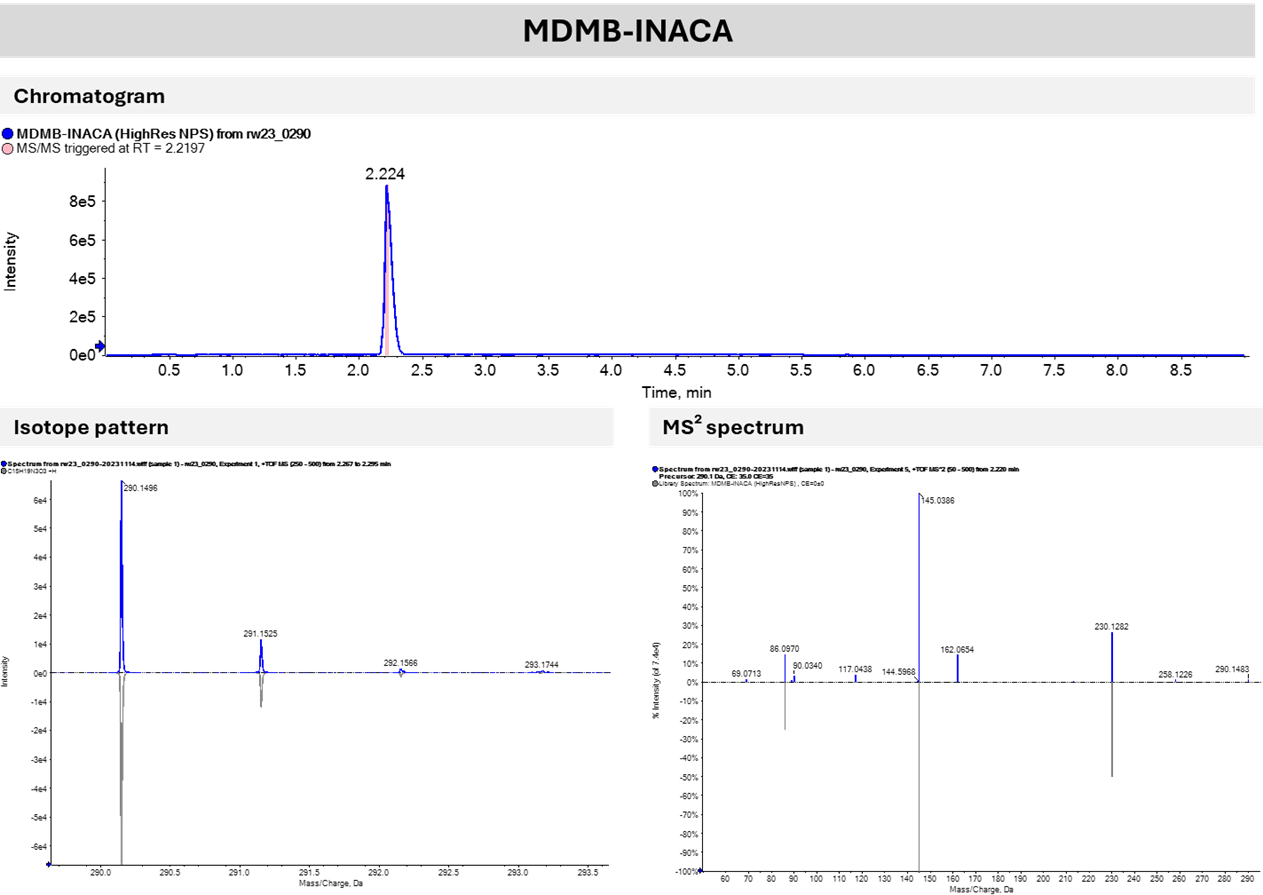


Figure S5.3: Identification of MDMB-INACA in sample FL23/0290 by LC-QToF-MS.

**Section 6: Full analytical data for samples of waxy- or putty-like material seized in English prisons**

Table S6.1: Full LC-QToF-MS data for samples of waxy- or putty-like materials seized in the English prisons. The MS was operated in all-ions mode with 3 collision energy scan segments at 0, 20, and 40 eV. Samples are arranged by seizure date and peak area. Details of the LC-QToF-MS analysis are provided, including retention time (RT) of the compound, exact mass ([M+H]^+^ (amu)), theoretical monoisotopic mass (amu), mass error (ppm), peak area, and MS fragments (amu) used to match compounds to HighResNPS database.

|  |  |  |  | **LC-QTOF-MS** | | | | | | **qNMR** |
| --- | --- | --- | --- | --- | --- | --- | --- | --- | --- | --- |
| **Sample ID** | **Seizure Date** | **Mass of**  **Material (g)** | **Analysis Result** | **RT (mins)** | **[M+H]^+^ (amu)** | **Theoretical Mass (amu)** | **Mass Error (ppm)** | **Peak Area** | **Fragments** | **% Composition in Sample by mass** |
| SP915A | 06/03/2023 | 11.0296 | MDMB-4en-PINACA | 4.456 | 358.2132 | 358.2125 | 1.67 | 41035842 | 213.1023; 298.1915; 145.0398; 171.0551 | 85 |
|  |  |  | MDMB-INACA | 2.823 | 290.1503 | 290.1499 | 1.44 | 2382014 | 145.0399; 230.1294; 86.0963 | 7 |
|  |  |  | 4F-MDMB-BUTINACA | 4.092 | 364.2033 | 364.2031 | 0.77 | 225781 | 219.0929; 304.1823; 145.0396 | 0^a^ |
|  |  |  | DMF |  |  |  |  |  |  | Low^b^ |
| SP915B | 06/03/2023 | 12.7091 | MDMB-4en-PINACA | 4.443 | 358.2133 | 358.2125 | 2.22 | 41644140 | 213.1026; 298.1917; 145.0398; 171.0558 | 82 |
|  |  |  | MDMB-INACA | 2.768 | 290.1503 | 290.1499 | 1.11 | 2546857 | 145.0399; 230.1291; 86.0963 | Low^b^ |
|  |  |  | 4F-MDMB-BUTINACA | 4.079 | 364.2035 | 364.2031 | 0.97 | 244500 | 219.0929; 304.1822; 145.0399 | 0^a^ |
|  |  |  | DMF |  |  |  |  |  |  | Low^b^ |
| SP916 | 24/04/2023 | 27.1308 | MDMB-4en-PINACA | 4.449 | 358.2127 | 358.2125 | 1.09 | 41540366 | 213.1024; 298.1914; 145.0395; 171.0553 | 80 |
|  |  |  | MDMB-INACA | 2.785 | 290.1503 | 290.1499 | 0.80 | 1663628 | 145.0399; 230.1289; 86.0962 | Low^b^ |
|  |  |  | DMF |  |  |  |  |  |  | 4 |
| SP917 | 16/06/2023 | 20.4823 | MDMB-4en-PINACA | 4.453 | 358.2128 | 358.2125 | 1.29 | 39436846 | 213.1024; 298.194; 145.0397; 171.0555 | 74 |
|  |  |  | MDMB-INACA | 2.778 | 290.1503 | 290.1499 | 0.88 | 2572943 | 145.0395; 230.1290; 86.0964 | 9 |
|  |  |  | DMF |  |  |  |  |  |  | 5 |
| SP918 | 11/07/2023 | 21.4202 | MDMB-4en-PINACA | 4.457 | 358.2129 | 358.2125 | 1.64 | 38090547 | 213.1025; 298.1917; 145.0400; 171.0554 | 74 |
|  |  |  | MDMB-INACA | 2.720 | 290.1502 | 290.1499 | 0.59 | 1930389 | 145.0395; 230.1290; 86.0964 | Low^b^ |
|  |  |  | DMF |  |  |  |  |  |  | 3 |
| SP919 | 11/07/2023 | 7.4730 | MDMB-4en-PINACA | 4.453 | 358.2127 | 358.2125 | 1.28 | 43016595 | 213.1024; 298.1914; 145.0398; 171.0558 | 81 |
|  |  |  | MDMB-INACA | 2.726 | 290.1504 | 290.1499 | 1.02 | 2991650 | 145.0396; 230.1291; 86.0964 | 7 |
|  |  |  | DMF |  |  |  |  |  |  | Low^b^ |
| SP920A | 29/08/2023 | 21.5613 | MDMB-4en-PINACA | 4.448 | 358.2125 | 358.2125 | 0.79 | 45695291 | 213.1023; 298.1912; 145.0397; 171.0553 | 38 |
|  |  |  | MDMB-INACA | 2.732 | 290.1501 | 290.1499 | 1.06 | 22315451 | 145.0397; 230.1290; 86.0964 | 26 |
|  |  |  | DMF |  |  |  |  |  |  | 7 |
| SP920B | 29/08/2023 | 23.2476 | MDMB-4en-PINACA | 4.451 | 358.2125 | 358.2125 | 0.76 | 45079927 | 213.1022; 298.1911; 145.0395; 171.0554 | 54 |
|  |  |  | MDMB-INACA | 2.744 | 290.1499 | 290.1499 | 0.64 | 21291952 | 145.0396; 230.1287; 86.0964 | 33 |
|  |  |  | DMF |  |  |  |  |  |  | 6 |
| SP921 | 29/08/2023 | 20.3666 | MDMB-4en-PINACA | 4.457 | 358.2125 | 358.2125 | 0.73 | 38997928 | 213.1022; 298.1913; 145.0396; 171.0551 | 52 |
|  |  |  | MDMB-INACA | 2.73 | 290.1498 | 290.1499 | -0.09 | 14737704 | 145.0397; 230.1288; 86.0965 | 35 |
|  |  |  | DMF |  |  |  |  |  |  | 3 |
| SP922A | 29/08/2023 | 23.5686 | MDMB-4en-PINACA | 4.46 | 358.2127 | 358.2125 | 0.93 | 38967156 | 213.1024; 298.1914; 145.0395; 171.0554 | 55 |
|  |  |  | MDMB-INACA | 2.816 | 290.1498 | 290.1499 | -0.33 | 14741247 | 145.0396; 230.1286; 86.0963 | 35 |
|  |  |  | DMF |  |  |  |  |  |  | 4 |
| SP922B | 29/08/2023 | 22.7653 | MDMB-4en-PINACA | 4.438 | 358.2128 | 358.2125 | 1.24 | 45122369 | 213.1024; 298.1915; 145.0397; 171.0554 | 21 |
|  |  |  | MDMB-INACA | 2.749 | 290.1499 | 290.1499 | 0.44 | 21571297 | 145.0396; 230.1287; 86.0963 | 18 |
|  |  |  | DMF |  |  |  |  |  |  | 2 |

^a^4F-MDMB-BUTINACA was identified in the LC-QTOF-MS analysis but was not visible in the NMR spectra for these samples.

^b^These compounds were identified in the NMR spectra, however the concentration was too low to quantify.

**Section 7: Examination photos of all samples of waxy- or putty-like materials seized in the Scottish and English prisons.**

FL23/0180-1


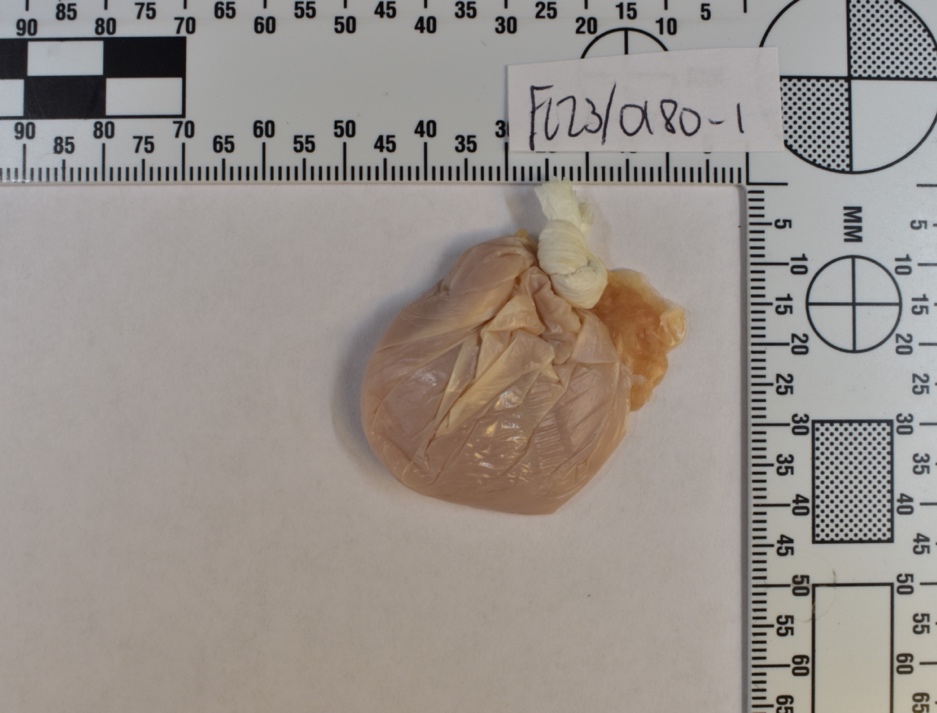

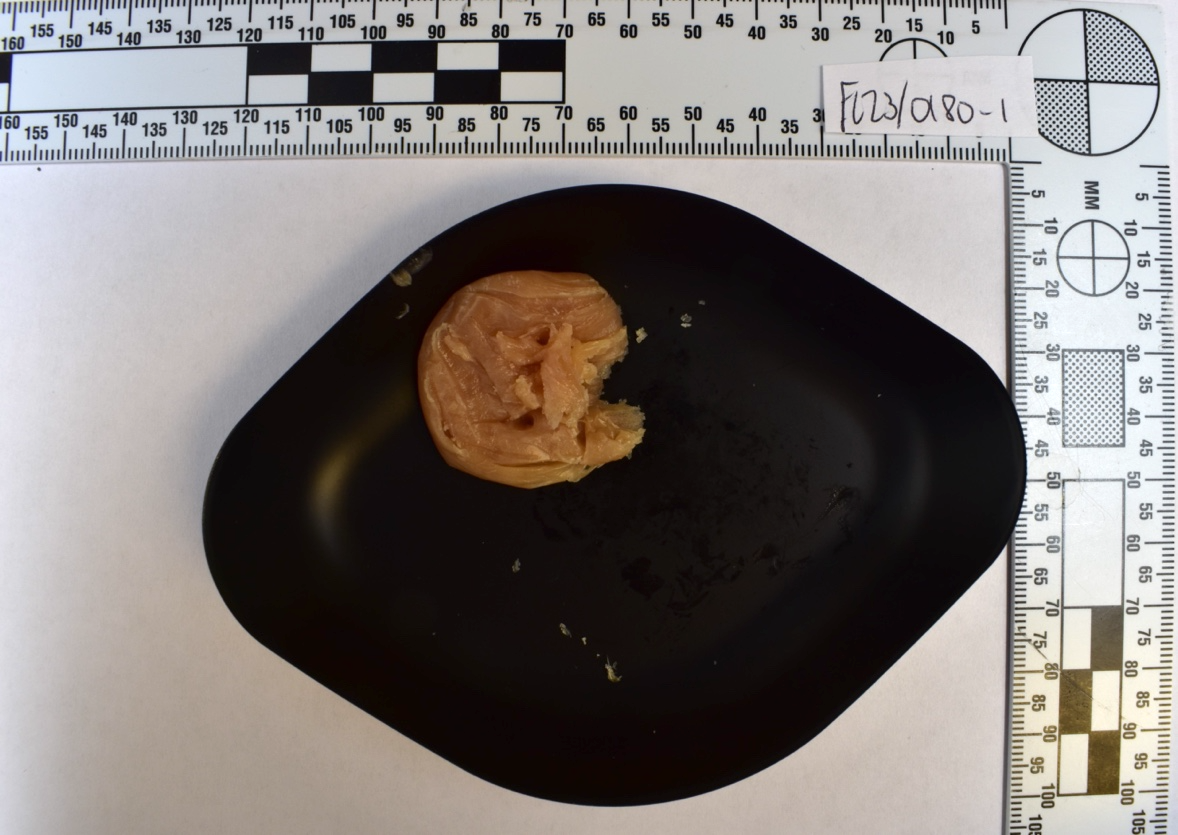

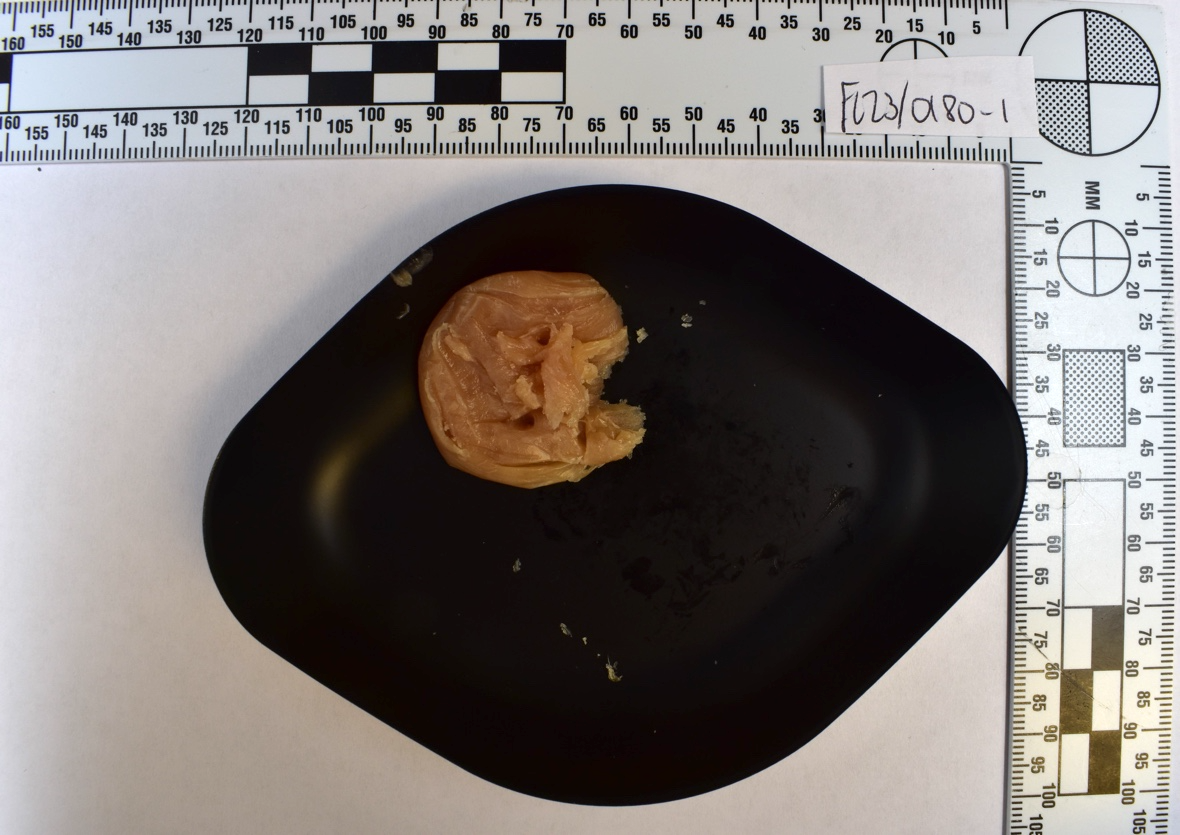

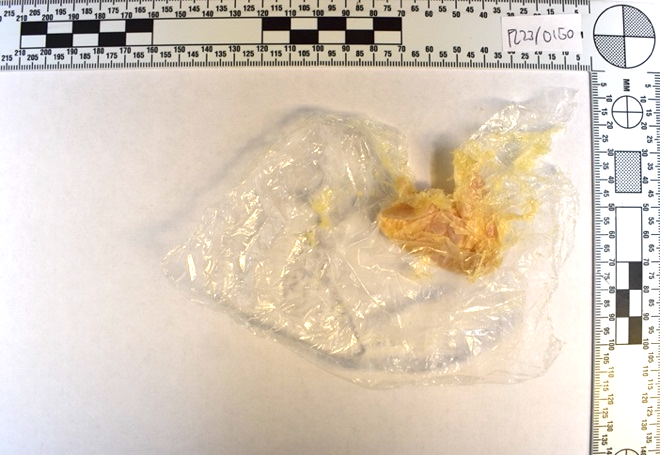


FL23/0150

FL23/0135

FL23/0174


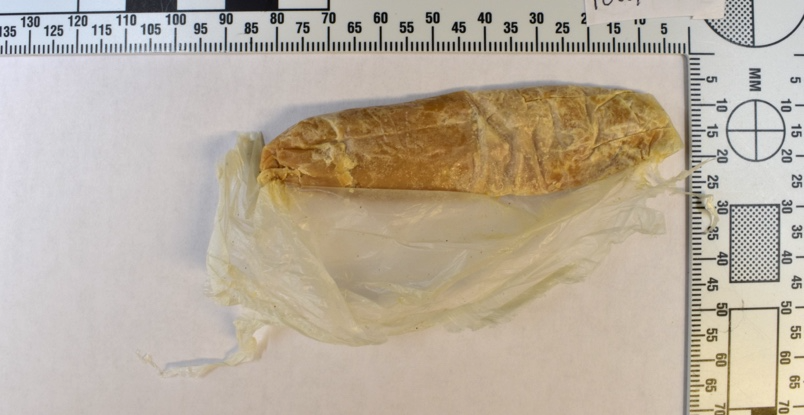

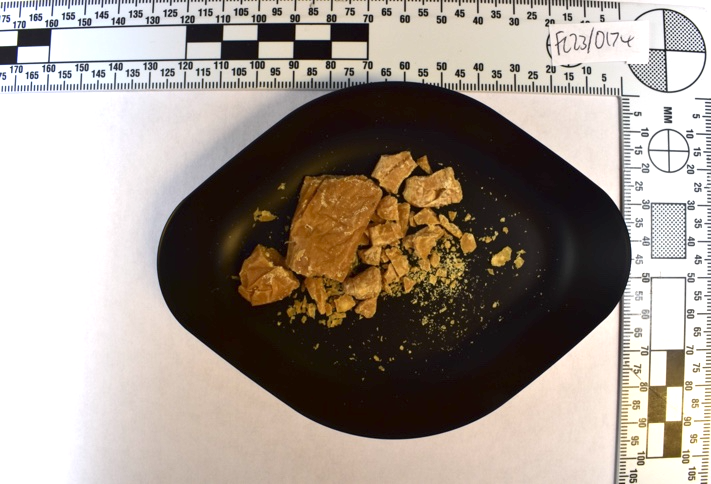

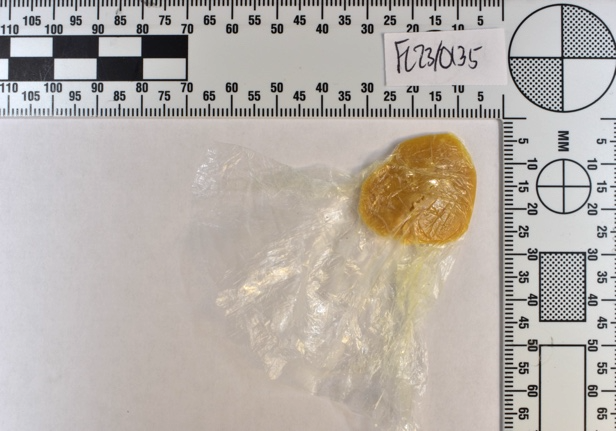


FL23/0199


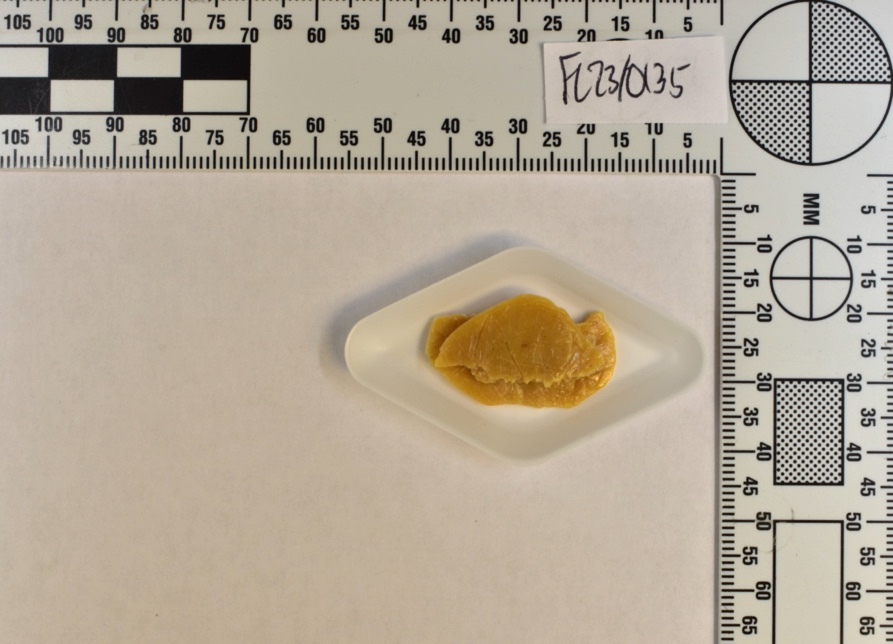


FL23/0188


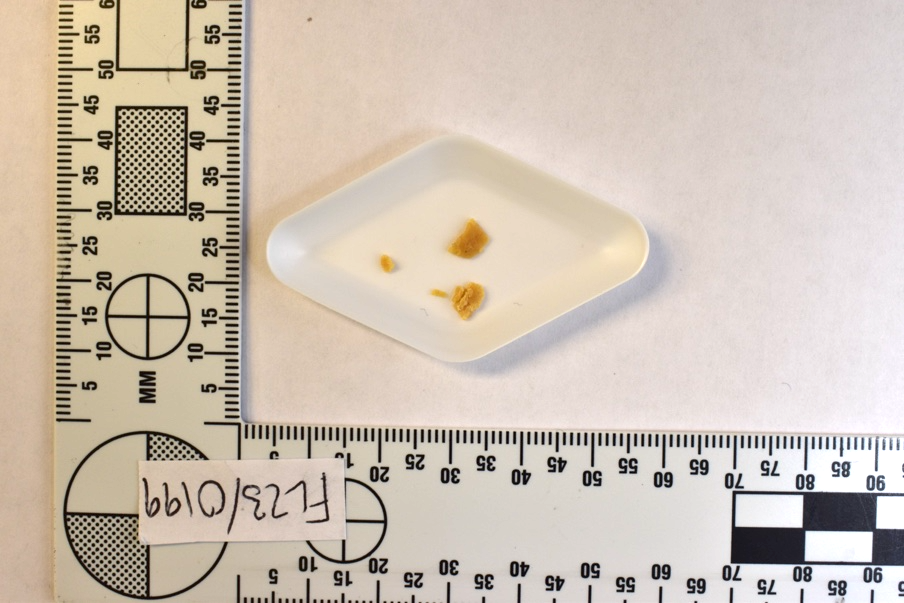

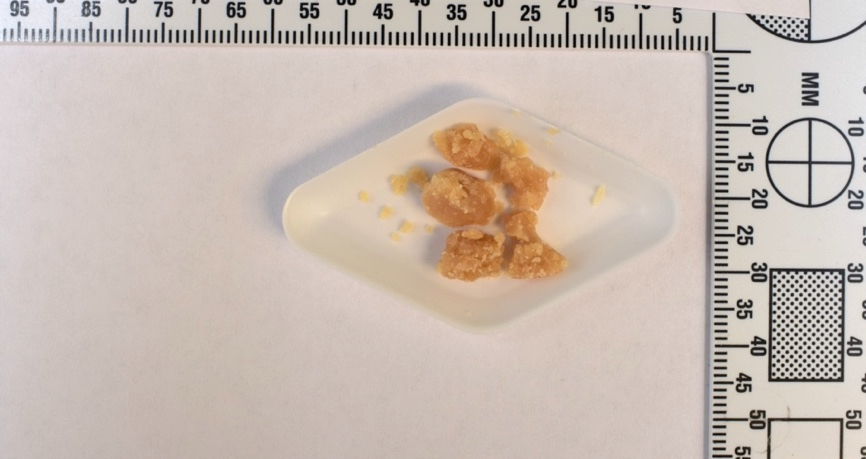


FL23/0367

FL23/0302

FL23/0301


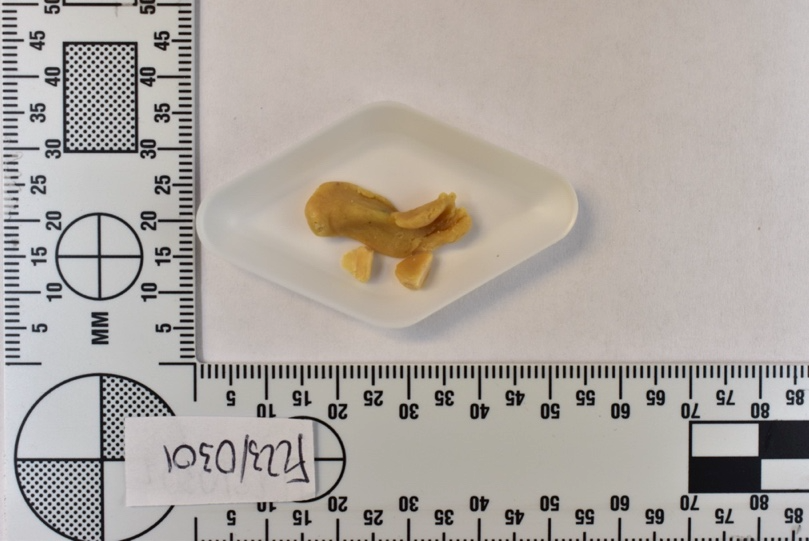

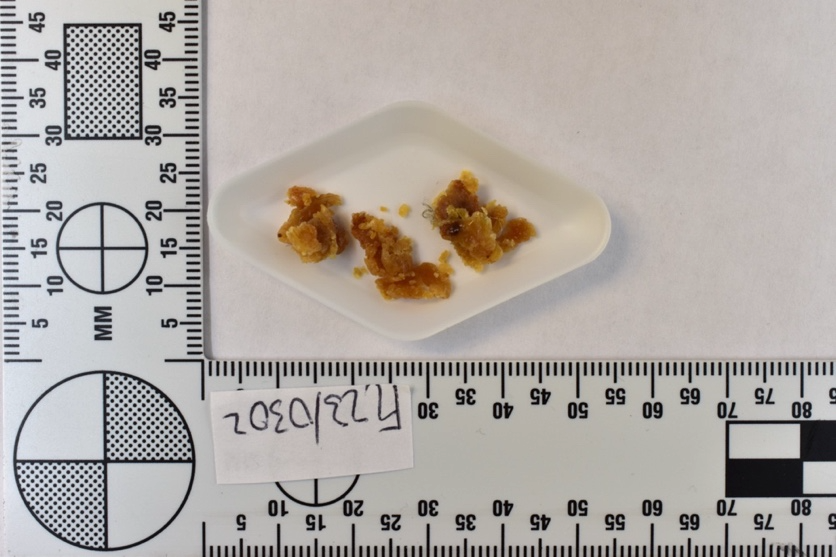

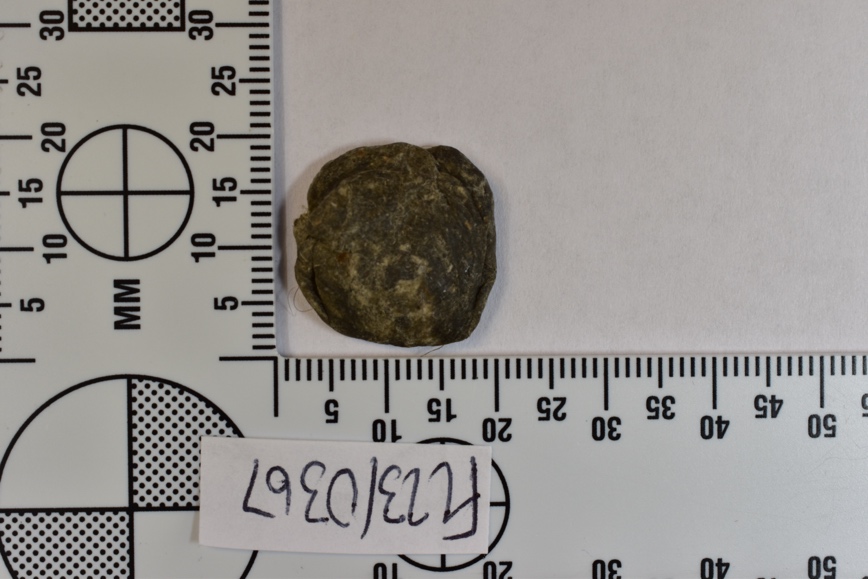


Figure S7.1: Examination photos of all samples of waxy- or putty-like materials seized from the Scottish prisons found to contain synthetic cannabinoids.

FL23/0152

FL23/0134


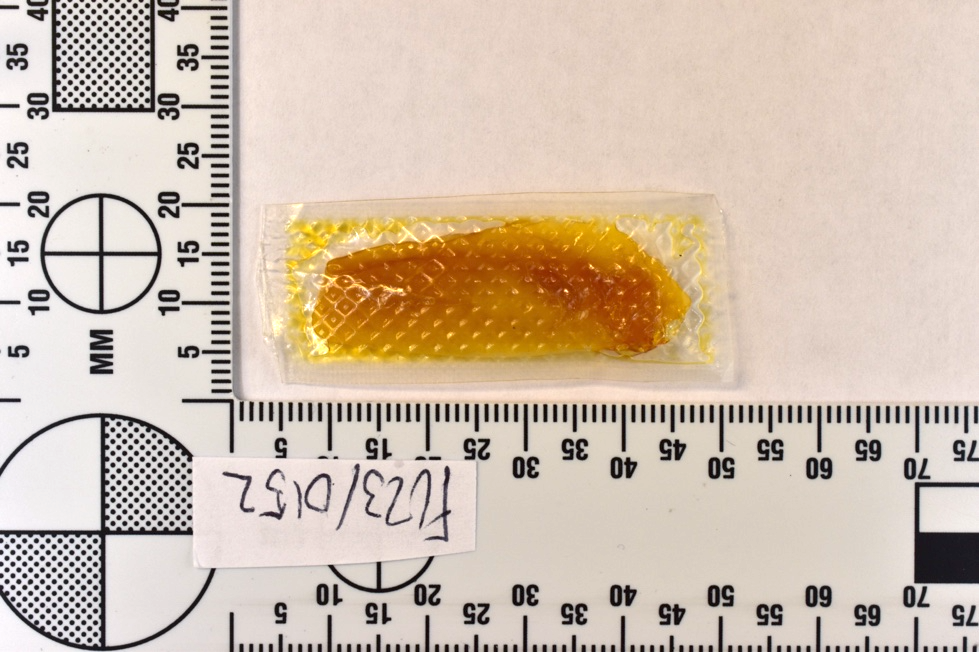

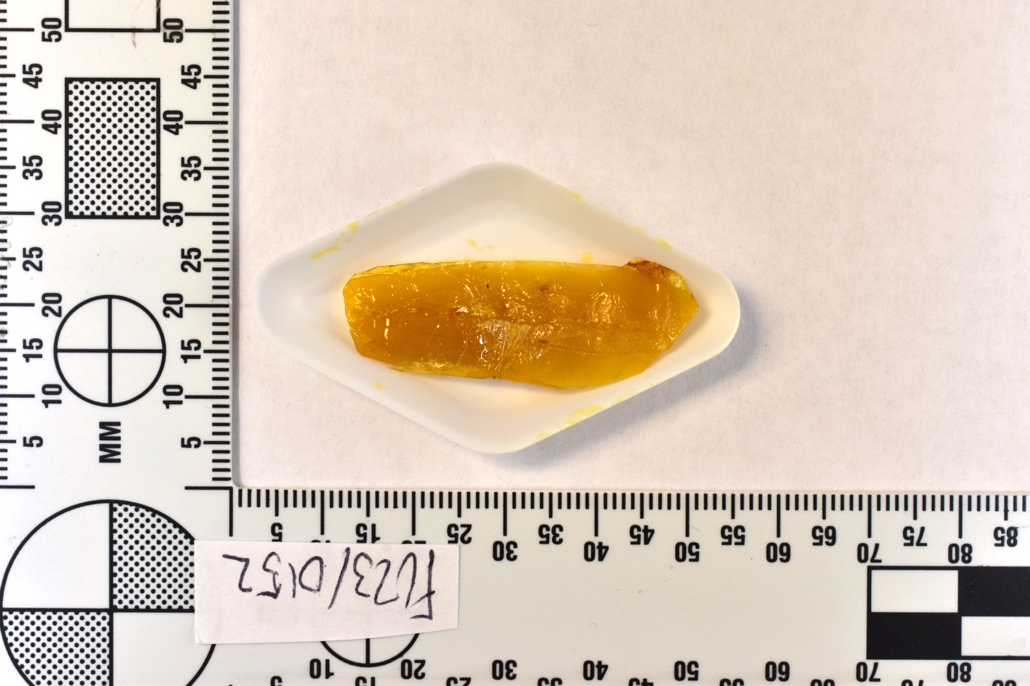

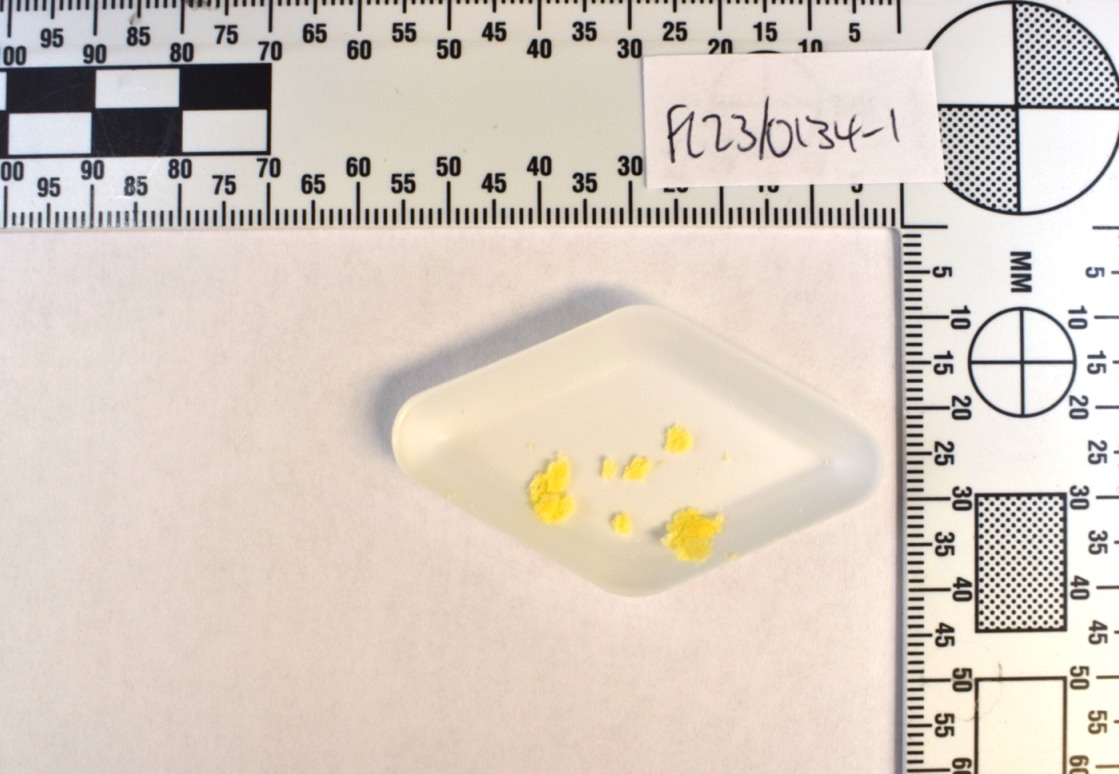


Figure S7.2: Examination photos of all samples of waxy- or putty-like materials seized from the Scottish prisons found to contain phytocannabinoids.

(b)

**
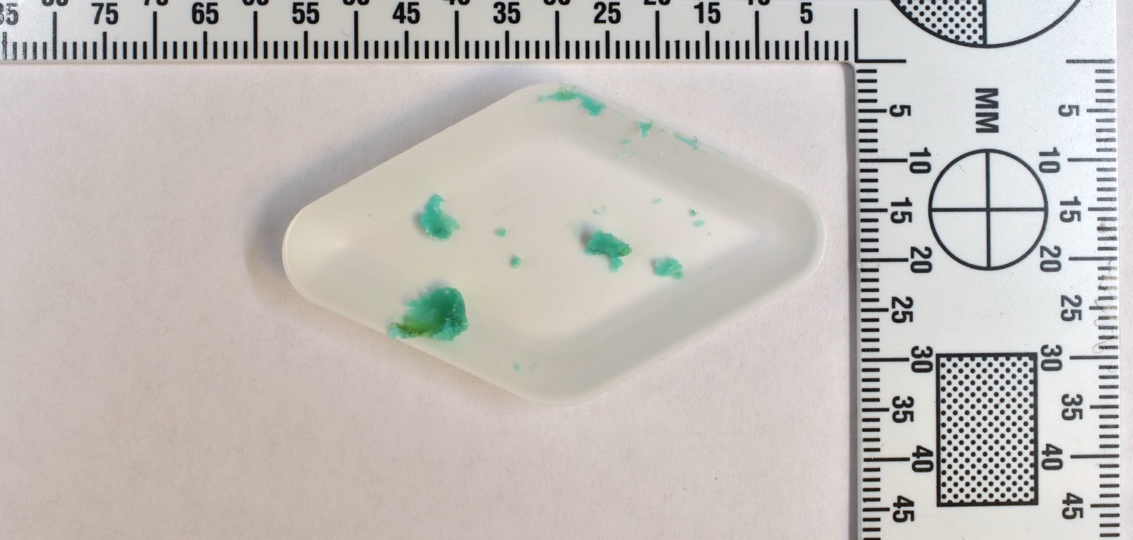
** **
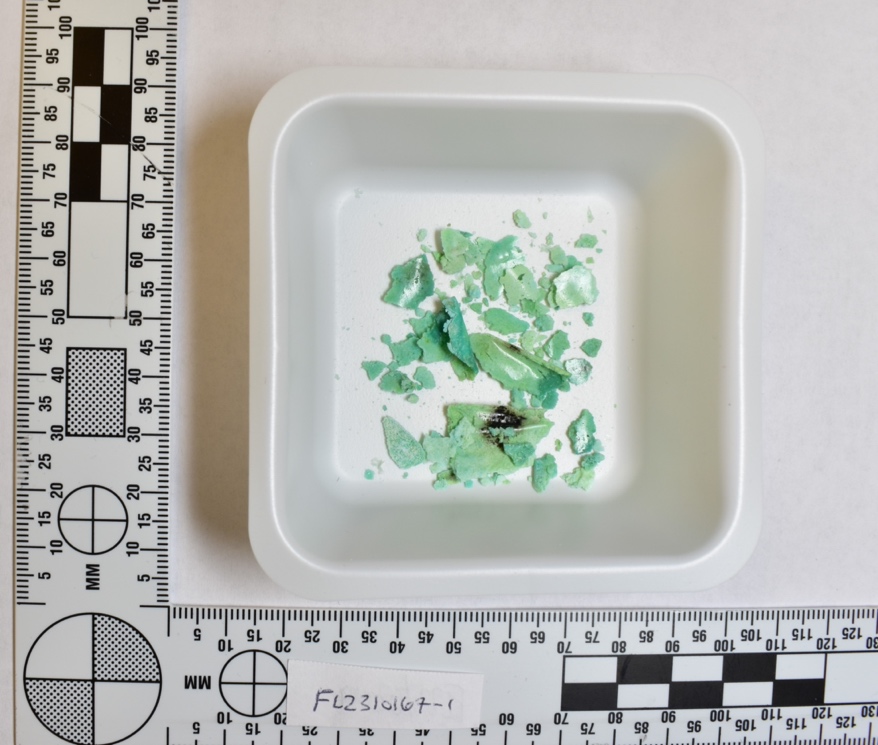
**

(a)

Figure S7.3: Examination photos of sample FL23/0167, a waxy- or putty-like material seized from the Scottish prisons found to contain gabapentinoids. (a) The material at first examination, following removal from evidence bag. (b). The material at a second examination, where the material had turned into a hard, “shatter”-like material, likely due to evaporation following exposure to air in an unsealed evidence bag.


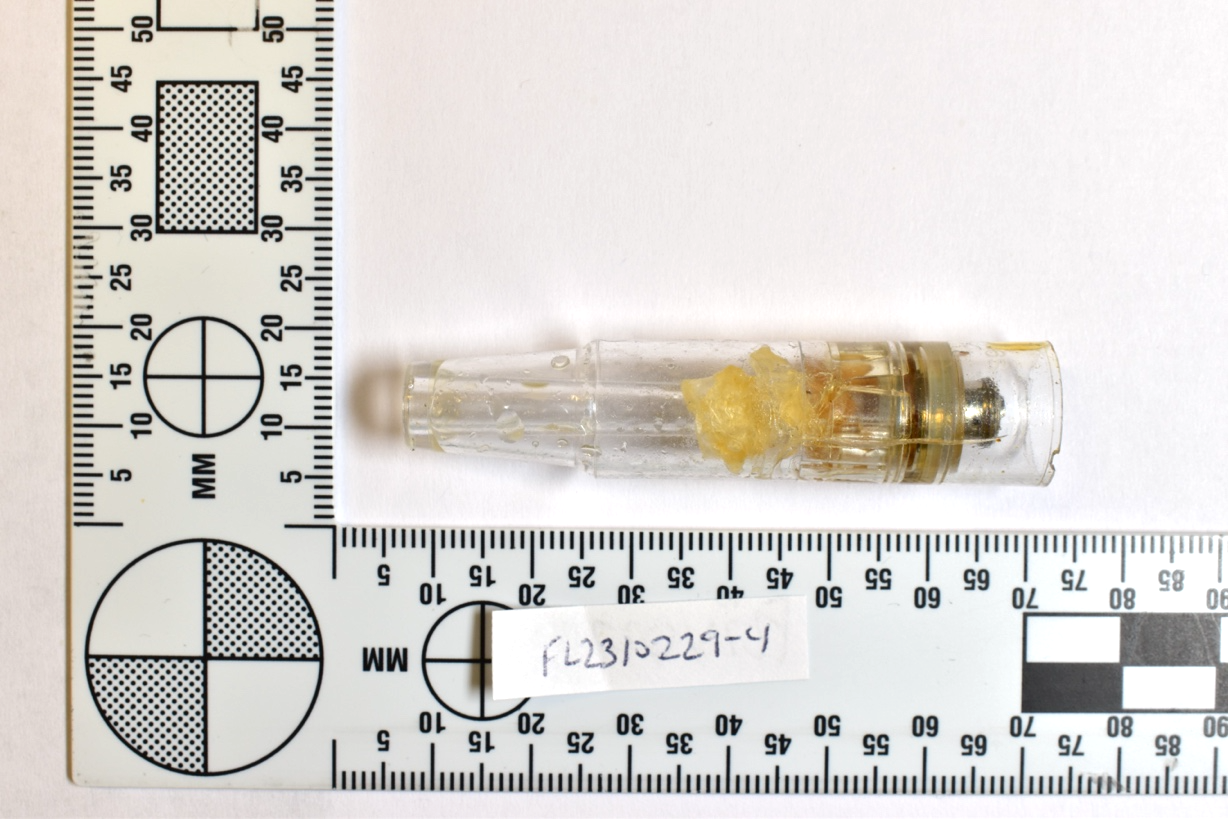

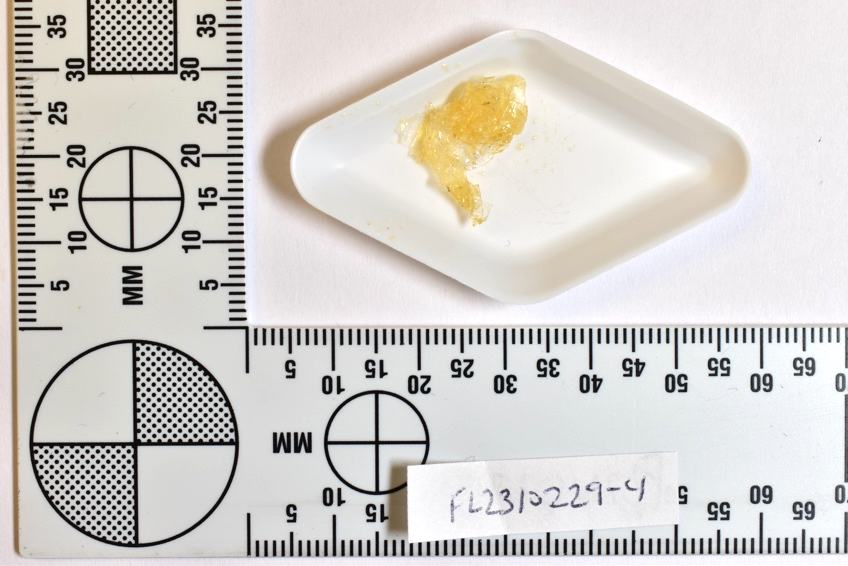


FL23/0229

FL23/0228


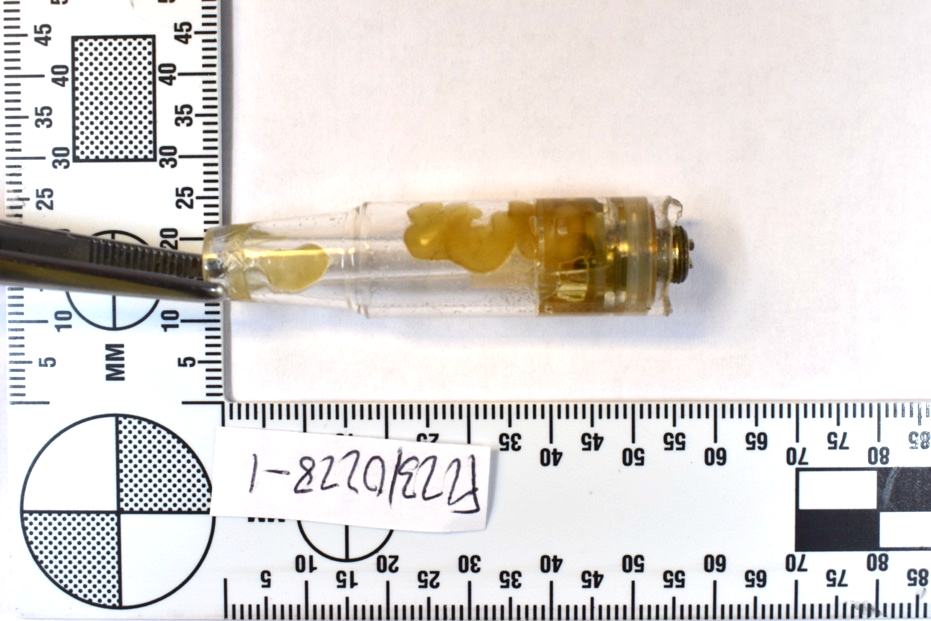

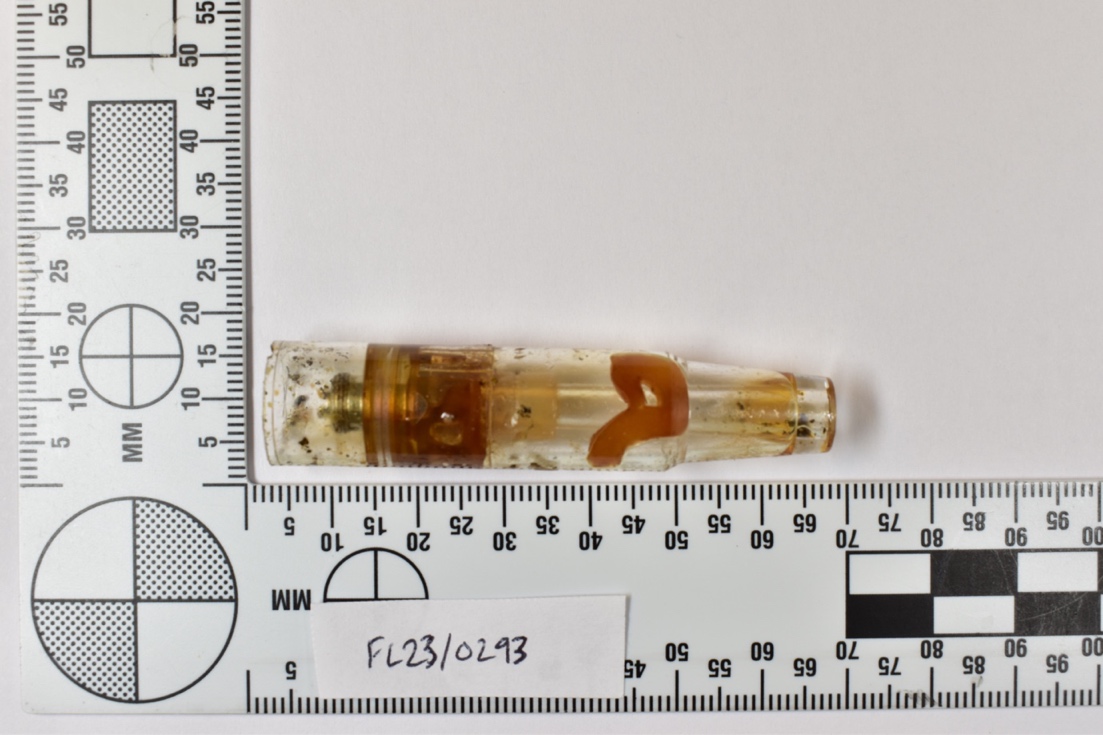


FL23/0293

FL23/0232


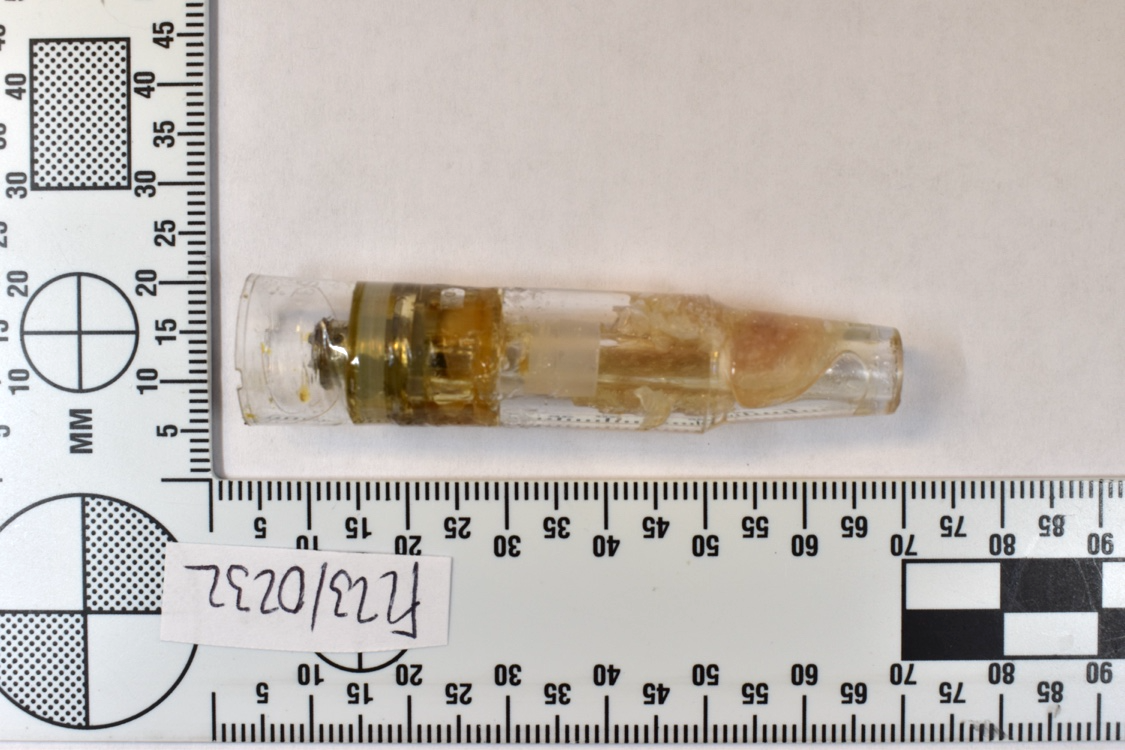

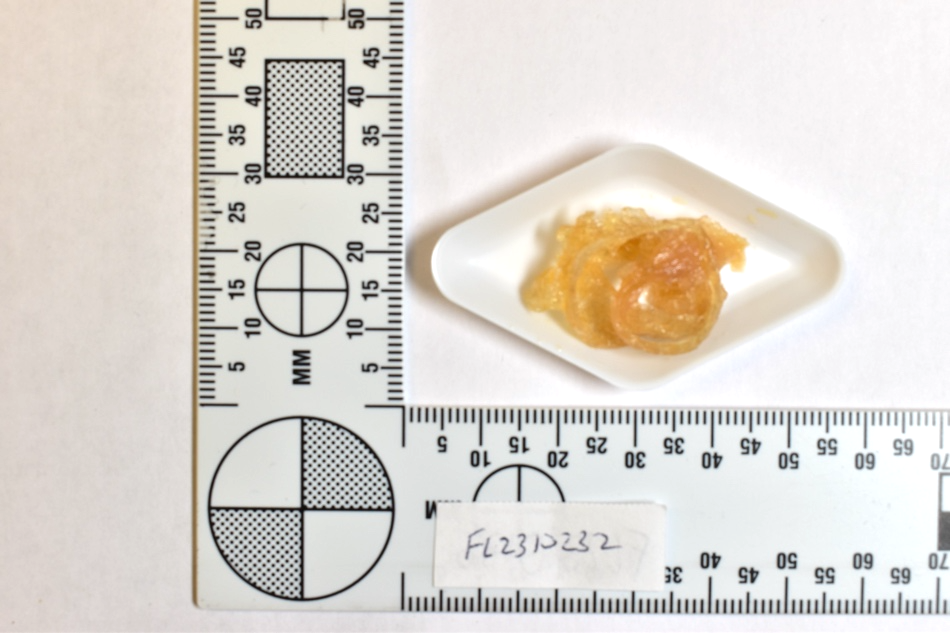


FL23/0291

FL23/0290


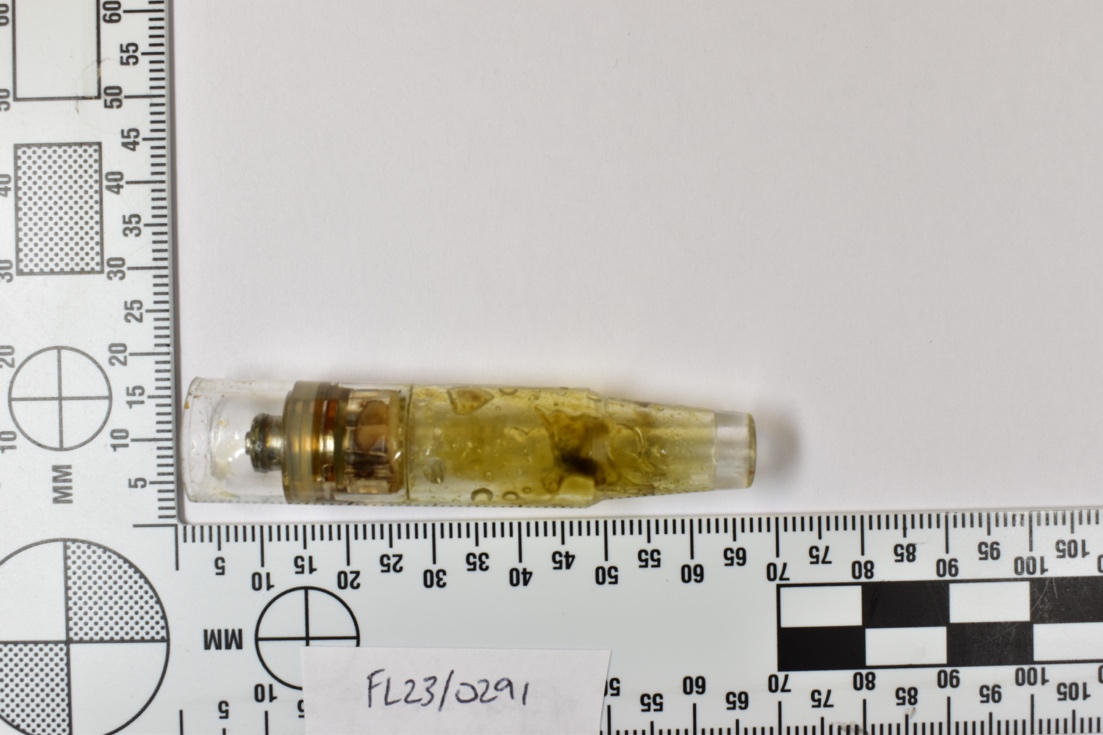


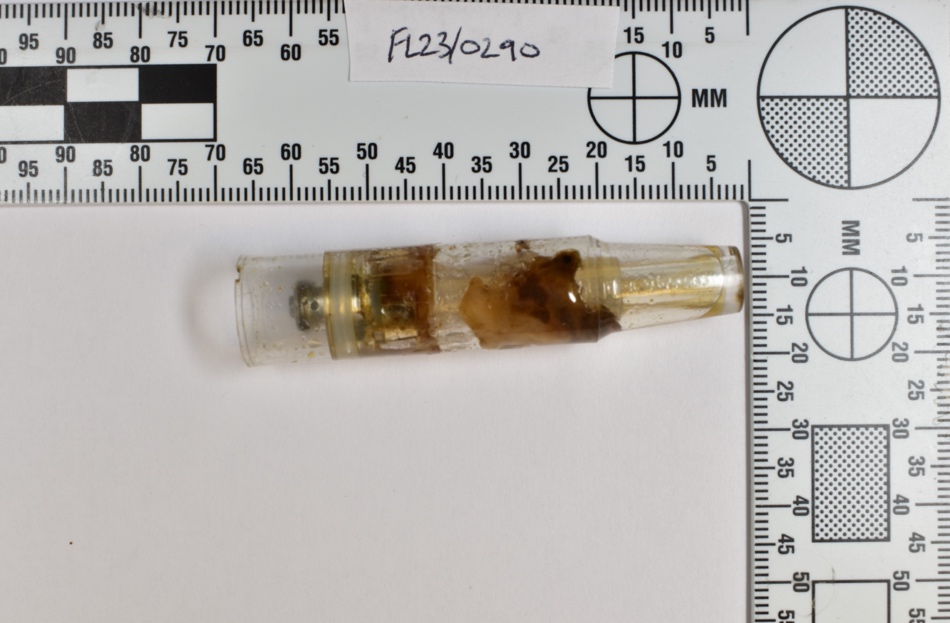


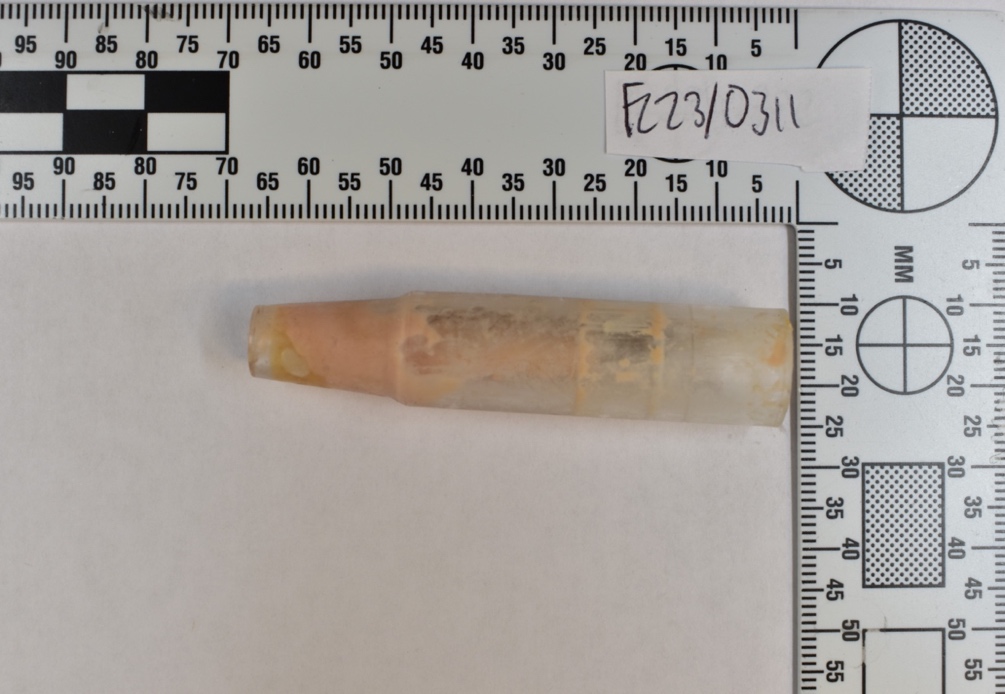

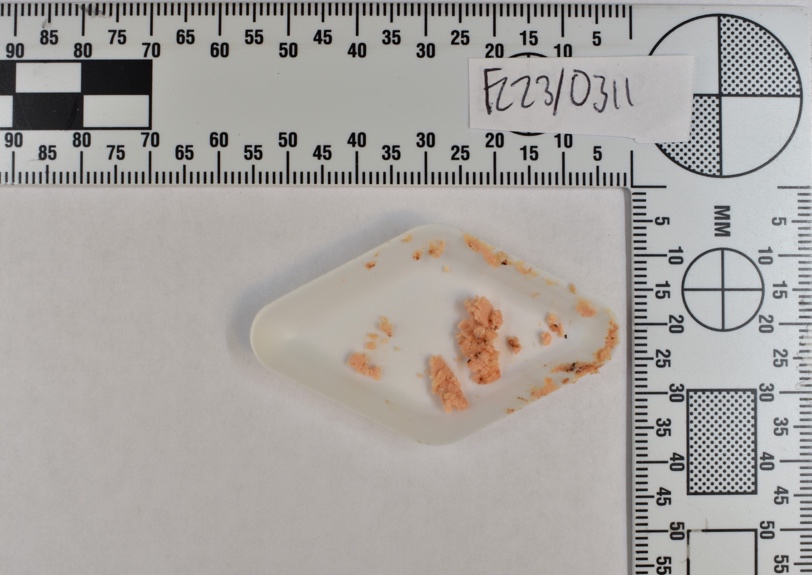

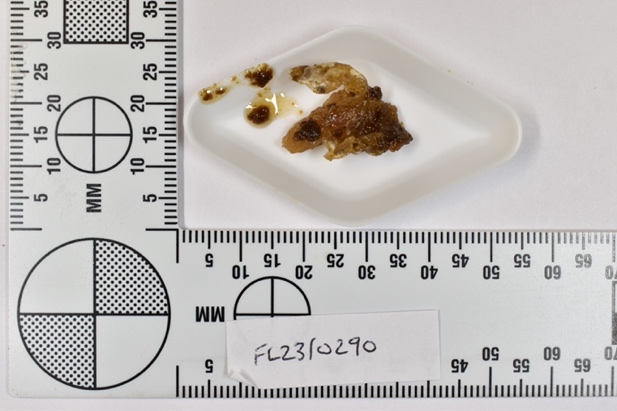


FL23/0311

Figure S7.4: Examination photos of all samples with e-cigarettes containing waxy- or putty-like materials seized from the Scottish prisons.


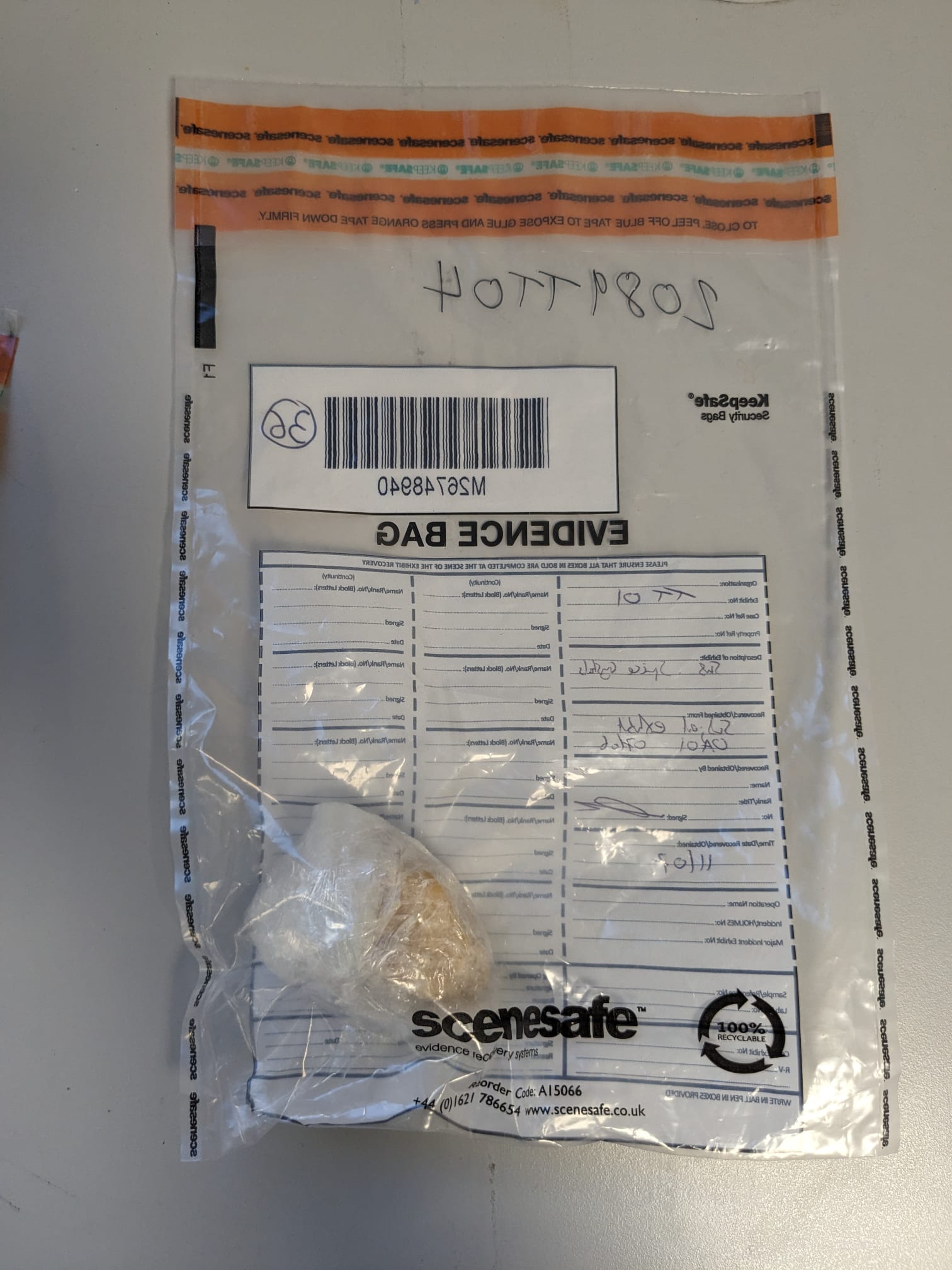


SP918


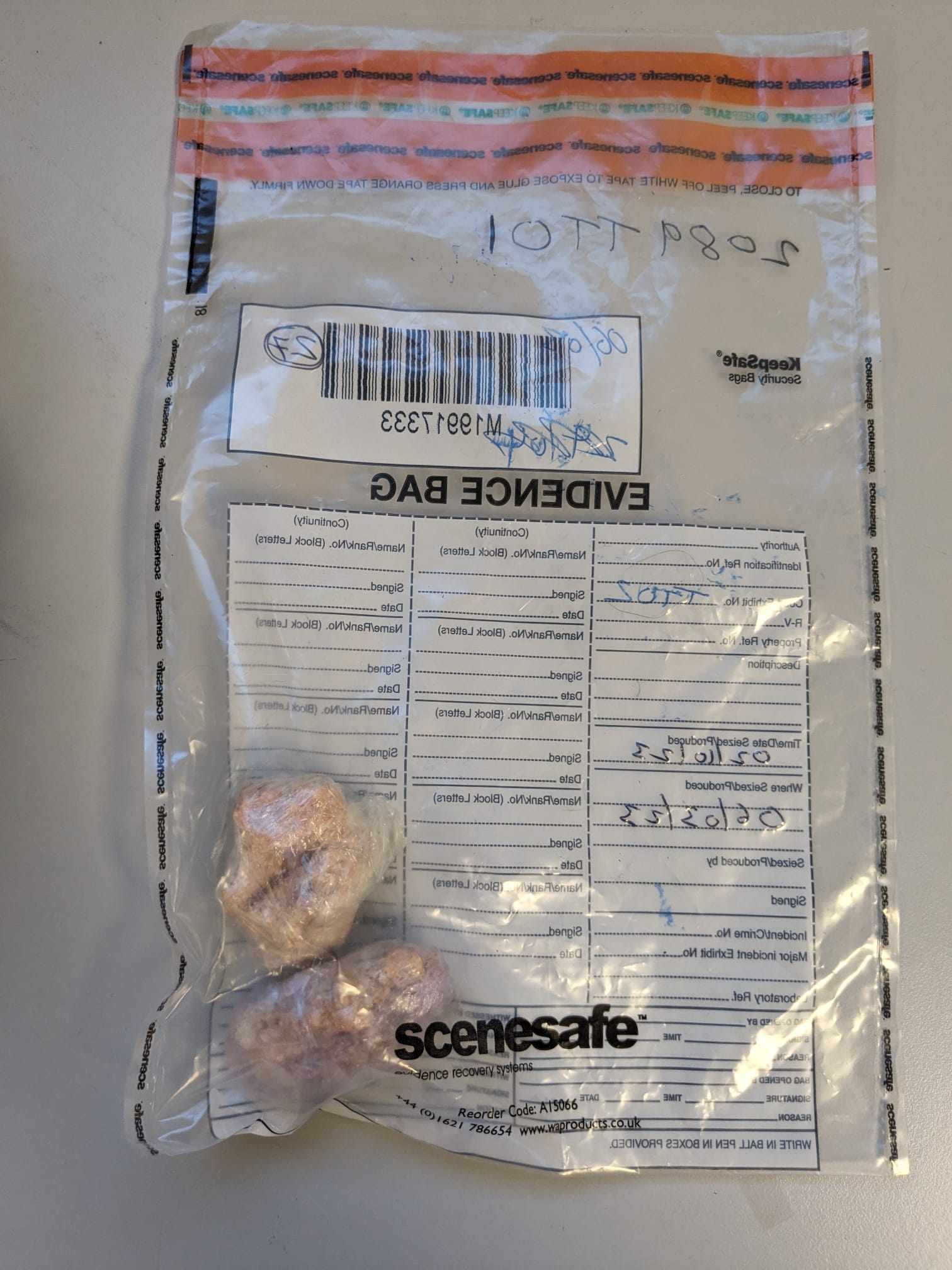


SP915


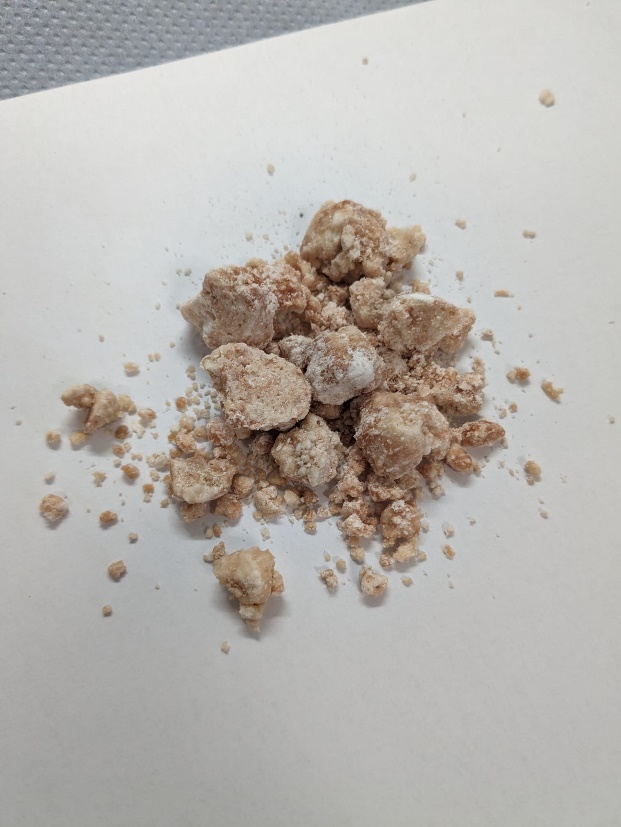

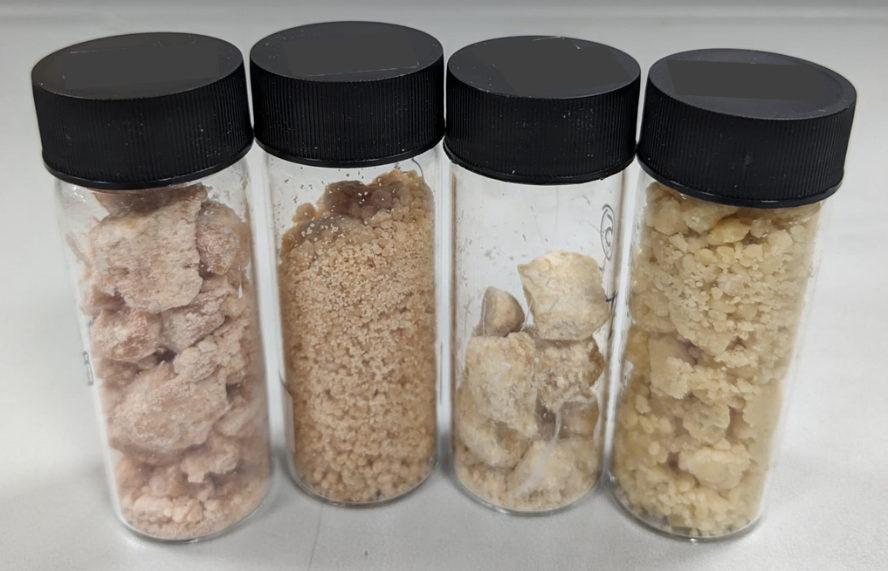


SP915

SP916

SP919

SP920

Figure S7.5: Examination photos of samples seized from the English prisons found to contain synthetic cannabinoids.
